# Transforming growth factor (TGF)-β1-induced miR-133a inhibits myofibroblast differentiation and pulmonary fibrosis

**DOI:** 10.1038/s41419-019-1873-x

**Published:** 2019-09-11

**Authors:** Peng Wei, Yan Xie, Peter W. Abel, Yapei Huang, Qin Ma, Linghai Li, Junfeng Hao, Dennis W. Wolff, Taotao Wei, Yaping Tu

**Affiliations:** 1 National Laboratory of Biomacromolecules, Institute of Biophysics, Chinese Academy of Sciences, Beijing, 100101 China; 2 Department of Pharmacology and Neuroscience, Creighton University School of Medicine, Omaha, NE 68178 USA; 3 University of Chinese Academy of Sciences, Beijing, 100049 China; 4 Key Laboratory of Genomics and Precision Medicine, Beijing Institute of Genomics, Chinese Academy of Sciences, Beijing, 100101 China; 5 Department of Anesthesiology, Beijing Chest Hospital, Capital Medical University, Beijing Tuberculosis and Thoracic Tumor Research Institute, Beijing, 101149 China; 6 Kansas City University of Medicine and Biosciences-Joplin, Joplin, MO 64804 USA

**Keywords:** Cell signalling, Respiratory tract diseases

## Abstract

Transforming growth factor (TGF)-β1, a main profibrogenic cytokine in the progression of idiopathic pulmonary fibrosis (IPF), induces differentiation of pulmonary fibroblasts to myofibroblasts that produce high levels of collagen, leading to concomitantly loss of lung elasticity and function. Recent studies implicate the importance of microRNAs (miRNAs) in IPF but their regulation and individual pathological roles remain largely unknown. We used both RNA sequencing and quantitative RT-PCR strategies to systematically study TGF-β1-induced alternations of miRNAs in human lung fibroblasts (HFL). Our data show that miR-133a was significantly upregulated by TGF-β1 in a time- and concentration-dependent manner. Surprisingly, miR-133a inhibits TGF-β1-induced myofibroblast differentiation whereas miR-133a inhibitor enhances TGF-β1-induced myofibroblast differentiation. Interestingly, quantitative proteomics analysis indicates that miR-133a attenuates myofibroblast differentiation via targeting multiple components of TGF-β1 profibrogenic pathways. Western blot analysis confirmed that miR-133a down-regulates TGF-β1-induced expression of classic myofibroblast differentiation markers such as ɑ-smooth muscle actin (ɑ-SMA), connective tissue growth factor (CTGF) and collagens. miRNA Target Searcher analysis and luciferase reporter assays indicate that TGF-β receptor 1, CTGF and collagen type 1-alpha1 (Col1a1) are direct targets of miR-133a. More importantly, miR-133a gene transferred into lung tissues ameliorated bleomycin-induced pulmonary fibrosis in mice. Together, our study identified TGF-β1-induced miR-133a as an anti-fibrotic factor. It functions as a feed-back negative regulator of TGF-β1 profibrogenic pathways. Thus, manipulations of miR-133a expression may provide a new therapeutic strategy to halt and perhaps even partially reverse the progression of IPF.

## Introduction

Idiopathic pulmonary fibrosis (IPF) is a chronic and fatal disease associated with deteriorating lung functioning, characterized by an excessive accumulation of extracellular matrix (ECM) proteins in response to chronic lung injury^1^. For patients with IPF, median survival time is 2–3 years from diagnosis. The pathological hallmarks of IPF include recruitment of inflammatory cells and excessive secretion of profibrotic cytokines such as transforming growth factor-β (TGF-β), aberrant fibroblast differentiation and proliferation, and persistence of apoptotic resistant myofibroblasts in the lesions^2,3^. TGF-β plays a central role in the pathogenesis of pulmonary fibrosis by promoting differentiation of fibroblasts into myofibroblasts that produce excessive extracellular matrix resulting in deteriorating lung function^4–7^. Treatment of fibroblasts with TGF-β leads to the phosphorylation and activation of Smad transcriptional factors that in turn result in the activation or repression of target genes including the differentiation marker genes α-smooth muscle actin (α-SMA), connective tissue growth factor (CTGF) and collagens^5^. In addition to this canonical Smad signaling pathway, non-canonical p38 mitogen-activated protein kinase (p38MAPK) signaling pathways also contribute to TGF-β-induced fibroblast differentiation^6,7^. Non-canonical signaling is context dependent and may have different effects in various cell types^5,8^.

MiRNAs emerged as a class of small RNAs that regulate whole networks of genes during different biological processes^9^. Several miRNAs such as miR-21, miR-218 and miR-29 have been shown to be dysregulated in fibrotic conditions^10–12^, and alterations in miRNA expression patterns may contribute to fibroblast differentiation^13^. Interestingly, several TGF-β-regulated miRNAs have been shown to modulate the TGF-β profibrogenic effects^10–12,14,15^. These multi-function/target miRNAs are of interest because their mimics and specific inhibitors are small and readily deliverable to manipulate differentiation potential in vivo. Thus, a complete definition of miRNA profiles is necessary to design specific drugs to target differentiation propensity during pulmonary fibrosis.

Systematic array approaches on mouse and human lung tissues have revealed a panel of miRNAs that are dysregulated in the process of pulmonary fibrogenesis^13,16^. However, since these studies were based on the analysis of whole lung miRNA pools, it is likely that some of miRNAs dysregulated in fibroblasts may not be detected by these approaches. In this study, using RNA sequencing and bioinformatics methods to analyze differentiated miRNAs expression between fibroblasts and TGF-β-induced myofibroblasts, we identified a novel TGF-β pathway desensitization mechanism mediated by a miR-133a-dependent negative feedback regulatory loop. More notably, ectopic miR-133a expression not only blocks TGF-β-induced differentiation of pulmonary fibroblasts into myofibroblasts, but also partially reverses this differentiated phenotype via targeting multiple components of TGF-β profibrogenic pathways, suggesting a potential novel therapeutic target to treat IPF.

## Results

### Identification of TGF-β1-induced miR-133a as a candidate that blocks pulmonary fibroblast differentiation

**Fig. 1:**
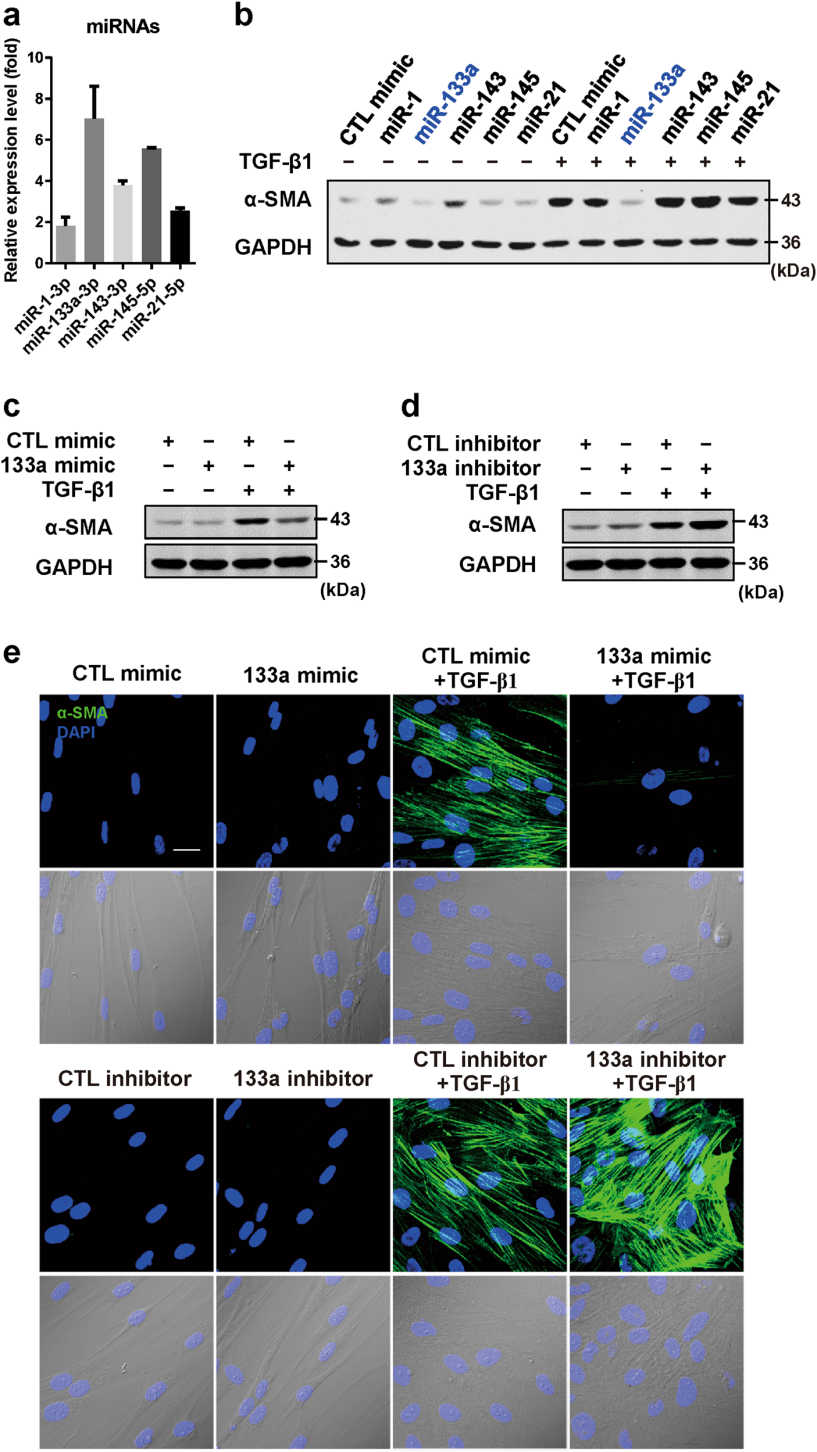
TGF-β1-induced miR-133a blocks pulmonary fibroblast differentiation. **a** RT-PCR analysis of TGF-β1-induced upregulation of miR-133, -1, -143, -145 and -21 in human lung fibroblast (HFL) cells. Cells were transfected with 30 nM of various miRNAs (**b**), miR-133a mimic (**c**), miR-133a inhibitor (**d**) or the control mimic (CTL) for 24 h, and then were stimulated without or with 1 ng/mL TGF-β1 for 48 h. α-SMA levels were analyzed by western blot and GAPDH was used as an internal control. Experiments were performed three times with similar results. **e** HFL cell differentiation was assessed by immunofluorescence staining for α-SMA. Up: representative staining images of α-SMA–positive stress fibers (green) and DAPI (blue) showing nuclei under confocal laser scanning microscopy. Down: bright field image and DAPI staining of nuclei (blue) (scale bar = 30 μm)

Since TGF-β-induced fibroblast differentiation is essential for pulmonary fibrosis progression, we conducted RNA sequence analysis of TGF-β1-treated human lung fibroblast (HFL) cells to identify upregulated miRNAs that may mediate TGF-β1-induced fibroblast differentiation. Compared to the control group, at least 30 miRNAs were upregulated in the TGF-β1-treated group by >2.3-fold (Table 1). Five upregulated miRNAs (miR-1, 21, 143, 145 and 133a) were verified by quantitative RT-PCR (Fig. 1a). We then transfected these miRNAs into HFL cells to investigate whether these TGF-β1-induced miRNAs modulate fibroblast differentiation into myofibroblasts, characterized by α-SMA expression^5,17^. Surprisingly, among the five selected miRNAs, miR-133a markedly attenuated TGF-β1-induced fibroblast differentiation as indicated by the reduction of TGF-β1-induced α-SMA protein expression (Fig. 1b). In contrast, miR-1 and miR-21 had little effects whereas miR-143 and miR-145 enhanced TGF-β1-induced fibroblast differentiation. Western blot analysis showed that transfection of miR-133a mimic attenuated TGF-β1-induced α-SMA expression (Fig. 1c) whereas a hairpin inhibitor of miR-133a enhanced TGF-β1-induced α-SMA expression in HFL cells (Fig. 1d). Immunostaining of α-SMA protein using its antibody showed that HFL cells treated with TGF-β1 displayed abundant stress fibers that stained intensely for α-SMA. MiR-133a mimic attenuated the TGF-β1-induced α-SMA-positive stress fibers whereas inhibition of endogenous miR-133a by its inhibitor enhanced the TGF-β1-induced α-SMA-positive stress fibers in HFL cells (Fig. 1e). These data suggest that TGF-β1-induced miR-133a may function as an anti-fibrotic factor, which provides a strong rationale for us to further perform mechanistic studies to determine the role of miR-133a in regulation of fibroblast differentiation and pulmonary fibrosis.

**Table 1:** microRNAs upregulated in TGF-β1-treated group (>2.3-fold) compared to untreated human primary fibroblasts

miRNA	Fold up-regulated, TGF-β1 vs. CTL
hsa-miR-143-5p	7.6
hsa-miR-145-5p	7.3
hsa-miR-145-3p	7.0
hsa-miR-143-3p	7.0
hsa-miR-1-3p	6.7
hsa-miR-181a-2-3p	5.9
hsa-miR-148a-5p	3.9
hsa-miR-148a-3p	3.8
hsa-miR-199b-5p	3.5
hsa-miR-2355-5p	3.5
hsa-miR-21-3p	3.4
hsa-miR-214-5p	3.2
hsa-miR-4448	3.1
hsa-miR-770-5p	3.1
hsa-miR-181b-2-3p	3.0
hsa-miR-6509-3p	3.0
hsa-miR-4532	2.9
hsa-miR-133a-3p	2.8
hsa-miR-6715a-3p	2.8
hsa-miR-6803-3p	2.6
hsa-miR-424-5p	2.6
hsa-miR-3182	2.6
hsa-miR-214-3p	2.5
hsa-miR-4700-3p	2.4
hsa-miR-548aw	2.4
hsa-miR-9-5p	2.4
hsa-miR-27b-3p	2.4
hsa-miR-23b-3p	2.4
hsa-miR-21-5p	2.4
hsa-miR-27b-5p	2.4

### TGF-β1 induces miR-133a expression via both Smad3 and p38MAPK signaling pathways

**Fig. 2:**
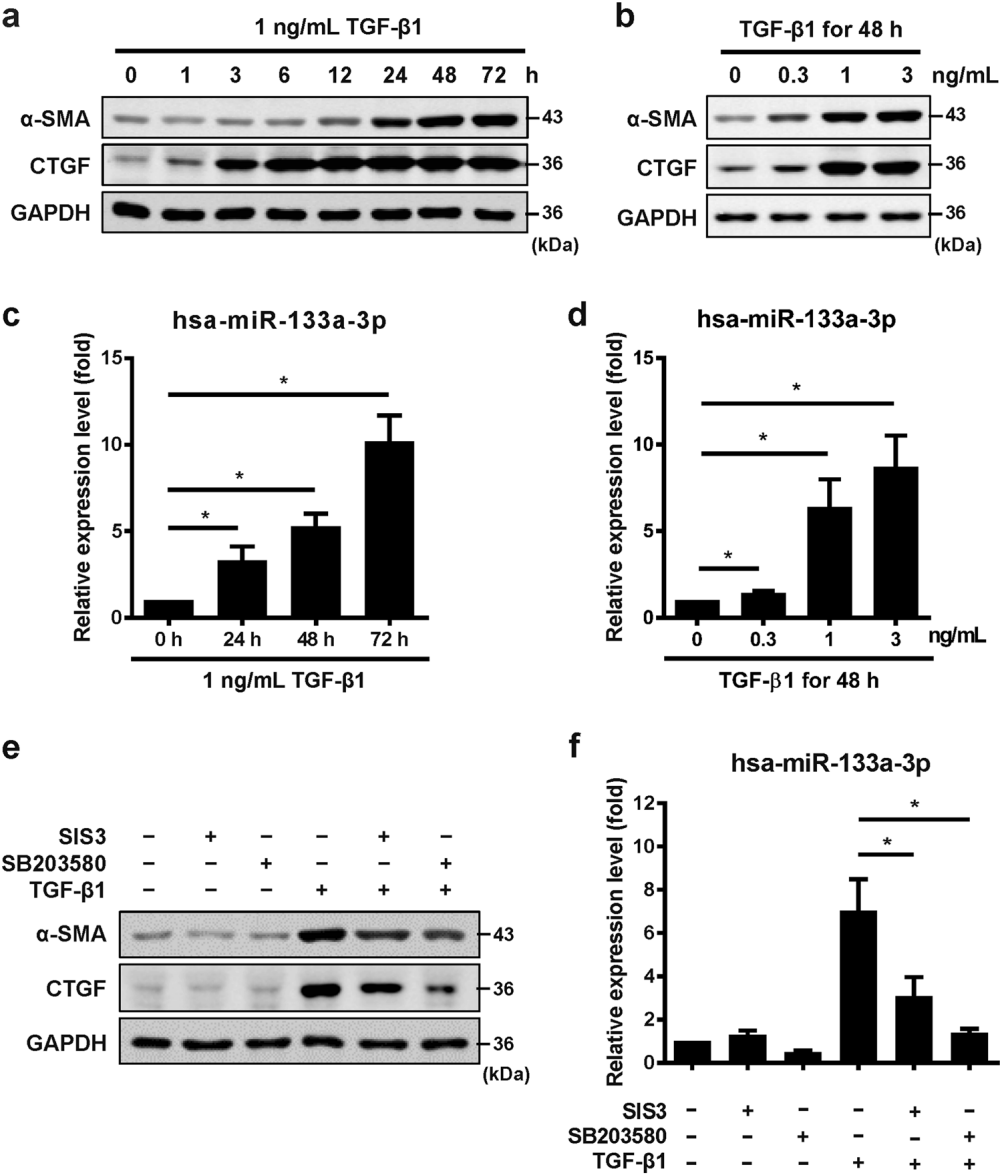
TGF-β1 induces miR-133a expression via both Smad3 and p38MAPK signaling pathways. HFL cells were stimulated with 1 ng/mL of TGF-β1 for different periods of time (**a**, **c**) or with various concentrations of TGF-β1 for 48 h (**b**, **d**). Cells were then harvested and subjected to western blot analysis of α-SMA and CTGF (**a**, **b**) protein expression or quantitative RT-PCR analysis of miR-133a expression (**c**, **d**). HFL cells were pretreated with 10 µM of Smad3 inhibitor (SIS3), p38MAPK inhibitor (SB203580) or vehicle (DMSO) for 30 min, and then stimulated with 1 ng/mL of TGF-β1 for 48 h. Cells were harvested and subjected to western blot analysis of α-SMA and CTGF protein expression (**e**) or quantitative RT-PCR analysis of miR-133a expression (**f**). Data are presented as the mean ± SEM. *P < 0.05 (*n* = 3–4)

Treatment of primary HFL cells with TGF-β1 induced a concentration- and time-dependent increase of myofibroblast differentiation markers, α-SMA and CTGF (Fig. 2a, b). Correlated with upregulation of α-SMA and CTGF proteins, quantitative RT-PCR showed a concentration and time-dependent increase of miR-133a expression (Fig. 2c, d). In contrast, stimulation with recombinant tumor necrosis factor (TNF) alpha had no effects on miR-133a expression in primary HFL cells (Supplementary Fig. 1).

TGF-β1 regulates gene transcriptions via activation of both canonical (Smad2/3) and non-canonical (p38MAPK) pathways^5,7^. Indeed, pretreatment of HFL cells with Smad3 inhibitor SIS3 or p38MAPK inhibitor SB203580 partially blocked TGF-β1-induced expression of α-SMA and CTGF (Fig. 2e). Interestingly, Smad3 and p38MAPK inhibitors also attenuated TGF-β1-induced miR-133a expression (Fig. 2f). Although SB203580 in concentration of 10 μM has potential inhibitory effects on the AKT signaling pathway^18,19^, pretreatment with 10 μM of LY294002, an inhibitor of the PI3K/AKT signaling pathway only slightly reduced miR-133a upregulation (Supplementary Fig. 2), suggesting that AKT signaling pathway may only play a limited role in TGF-β1-induced miR-133a upregulation.

### Quantitative proteomics analysis of miR-133a regulation of protein expression in TGF-β1-treated HFL cells

**Fig. 3:**
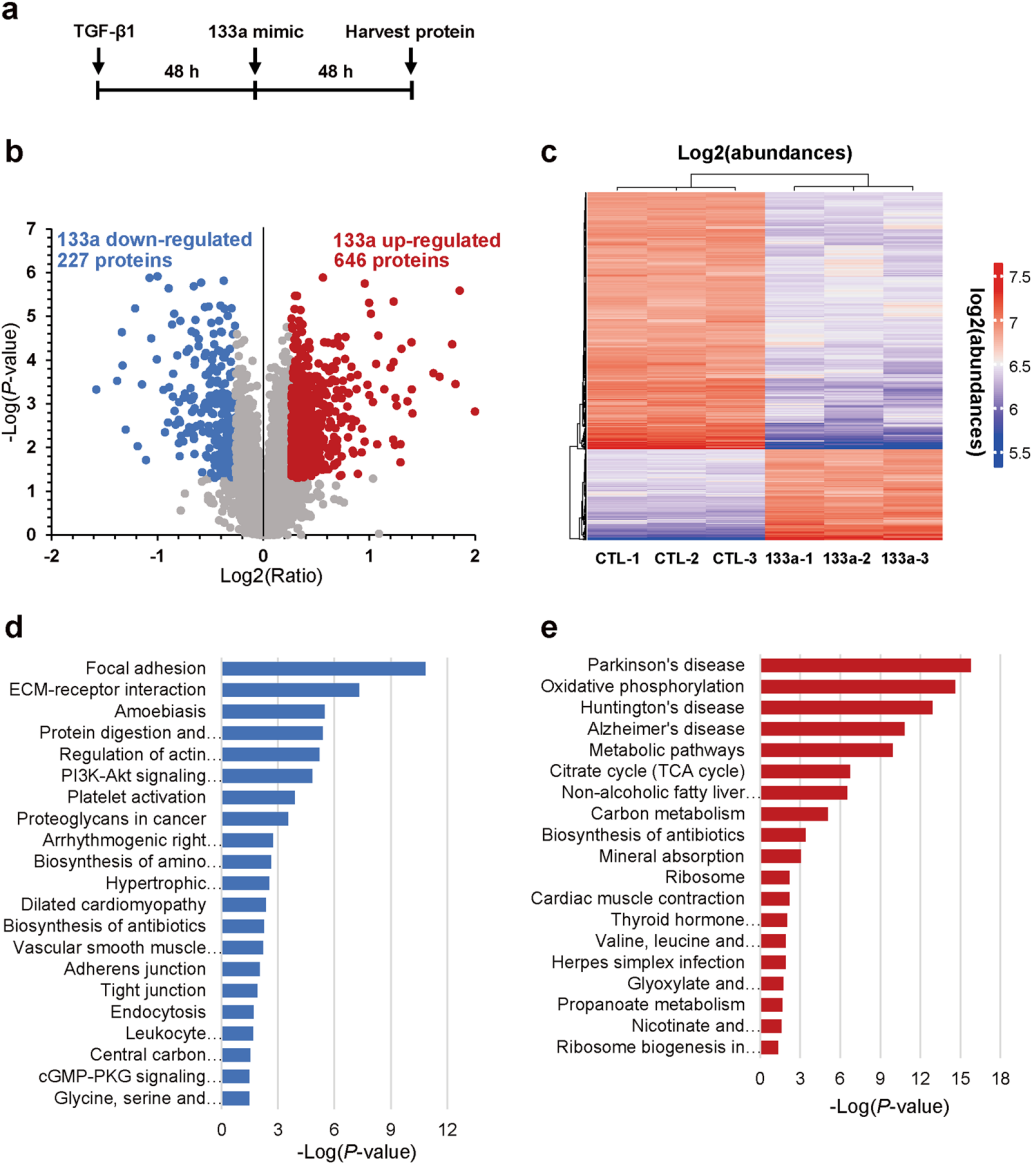
Quantitative proteomics and KEGG pathway enrichment analysis. **a** Schematic diagram of TGF-β1 and miR-133a mimic treatment. HFL cells were treated with 1 ng/mL of TGF-β1 for 48 h, and then transfected with miR-133a mimic or its control for an additional 48 h. **b** Volcano plot displays the overall proteins identified with a *P*-value < 0.05 and > 1.2-fold cutoff. In all, 227 proteins were down-regulated whereas 646 proteins were upregulated following miR-133a overexpression. **c** Overview of significantly upregulated or down-regulated proteins in three miR-133a overexpressed groups (133a-1, -2, -3) as compared to three control groups (CTL-1, -2, -3). The heatmap represents the log2 (abundances) of the differentially expressed genes in different samples. The color key from blue to red represents the log2(abundances) from low to high. **d** KEGG pathway analysis of down-regulated proteins. **e** KEGG pathway analysis of upregulated proteins. The vertical axis represents the pathway category and the horizontal axis represents the enrichment score [−log(*P*-value)] of the pathway. Significantly enriched KEGG pathways (*P* < 0.05) are presented. The data were analyzed by DAVID bioinformatics tools

Our data showed that miR-133a inhibits TGF-β1-induced fibroblast differentiation. We therefore tried to identify unique patterns of protein expression associated with miR-133a expression in myofibroblasts. HFL cells were stimulated with TGF-β1 for 48 h to induce differentiation, then transfected with miR-133a mimic or its control mimic for an additional 48 h (Fig. 3a). TMT-based quantitative proteomics analysis was performed to identify differentially expressed proteins between miR-133a transfected cells and the control group. A total of 5394 proteins were detected and quantified. By taking a *P*-value < 0.05 and fold change >1.2 as the cutoff, a total of 873 differentially expressed proteins were identified, including 227 down-regulated and 646 upregulated proteins in miR-133a transfected cells (Fig. 3b, c). To elucidate the functional roles of these differentially expressed proteins, we performed KEGG pathway enrichment analysis using the DAVID online analysis database (available online: https://david.ncifcrf.gov/). This analysis revealed that the pathways down-regulated in miR-133a transfected cells are related to focal adhesion, ECM-receptor interaction, adherens, and tight junctions (Fig. 3d). In contrast, pathways upregulated in miR-133a transfected cells presented a signature related to energy metabolism (Fig. 3e).

**Fig. 4:**
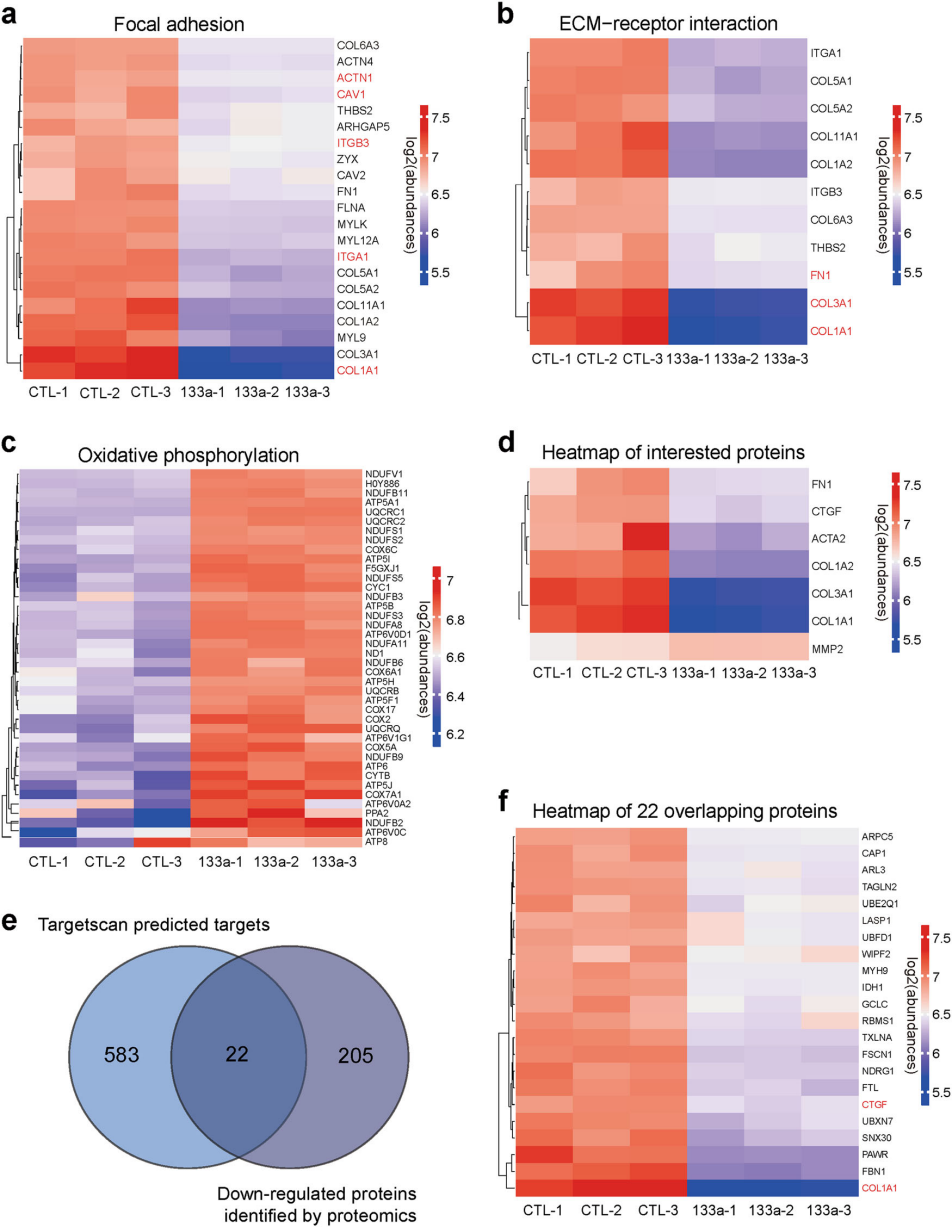
Overexpression of miR-133a regulates multiple pathways in TGF-β1-treated HFL cells. **a**–**d** Heatmap representing the log2 (abundances) of the differentially expressed genes in the selected KEGG pathways in miR-133a overexpressed HFL cells as compared to the CTL groups. The color key from blue to red represents the log2 (abundances) from low to high. **e** The overlap between Targetscan predicted miR-133a targets and miR-133a-down-regulated proteins identified by quantitative proteomics. **f** 22 overlapping proteins were analyzed and shown as Heatmap clusters

We further analyzed these pathways in detail. Focal adhesion related proteins ACTN1, CAV1, ITGB3, ITGA1 and COL1A1 and ECM-receptor interaction related proteins COL1A1, COL3A1 and FN1 were significantly down-regulated in miR-133a transfected cells (Fig. 4a, b). Energy metabolism-related mitochondria proteins NDUFV1, ATP5A1, UQCRC2, NDUFA8 and COX2 were also upregulated in miR-133a transfected cells (Fig. 4c). We also observed significant down-regulation of fibroblast differentiation marker proteins (Fig. 4d). Combined with Targetscan prediction of miR-133a putative targets, we found 22 overlapping targets among two groups including myofibroblast markers CTGF, COL1A1 that are down-stream effectors of TGF-β1 profibrogenic signal pathways (Fig. 4e, f).

### Overexpression of miR-133a alters the phenotype of TGF-β1-treated HFL cells

**Fig. 5:**
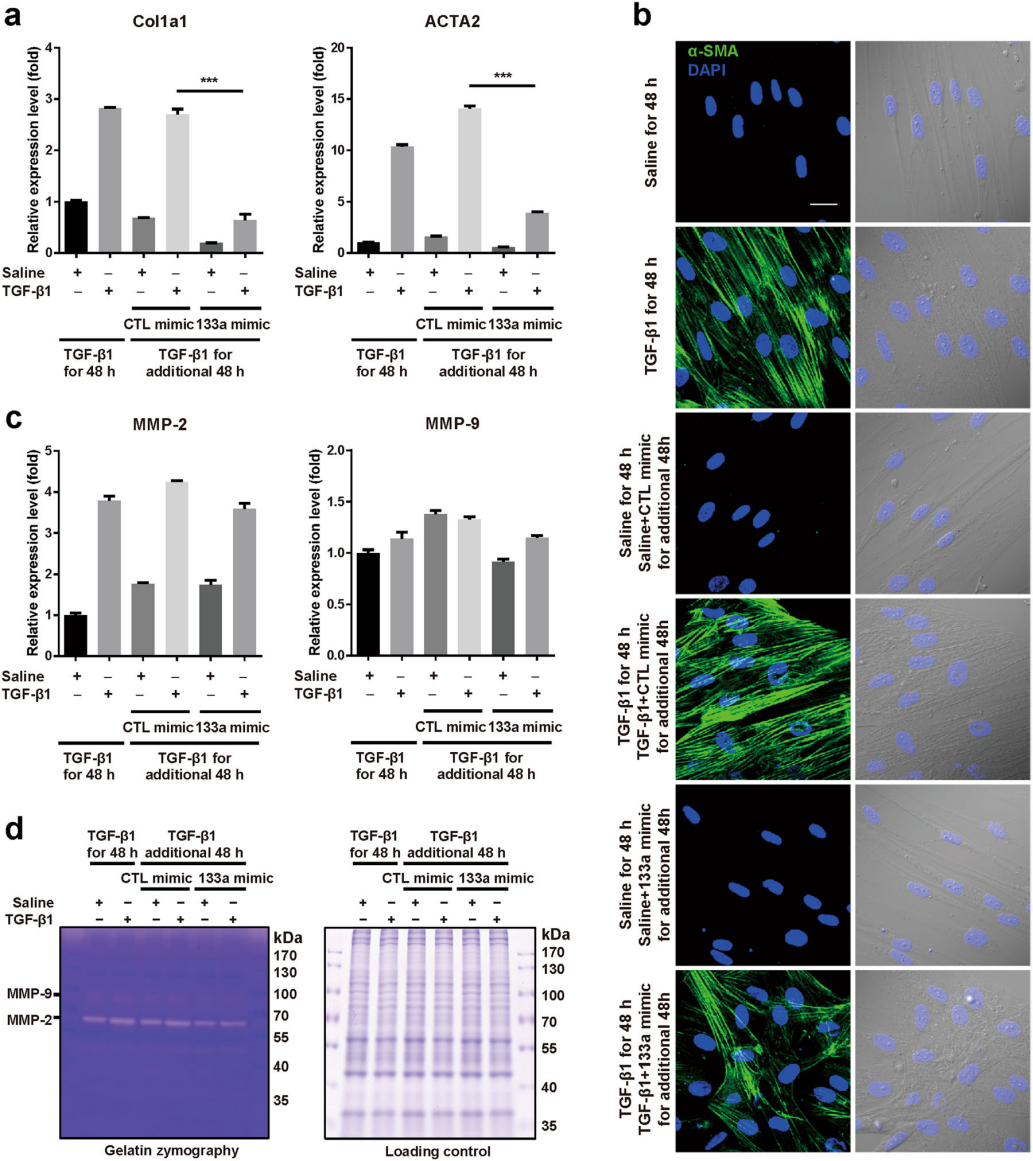
Overexpression of miR-133a alters the phenotype of TGF-β1-treated HFL cells. HFL cells were treated with 1 ng/mL of TGF-β1 for 48 h, and then transfected with miR-133a mimic or its control (CTL) for an additional 48 h. **a** Total RNAs were harvested and subjected to quantitative RT-PCR analysis of Col1a1 and ACTA2 mRNA expression. Data are mean ± SEM (*n* = 3) with ***P *<* 0.001. **b** HFL cell differentiation was assessed by immunofluorescence staining for α-SMA. Left: representative staining images of α-SMA–positive stress fibers (green) and DAPI (blue) showing nuclei under confocal laser scanning microscopy. Right: bright field image and DAPI staining of nuclei (blue) (scale bar = 30 μm). **c** Quantitative RT-PCR analysis of MMP-2 and MMP-9 mRNA expression. **d** Culture media were collected and run on 10% SDS-PAGE contains 1% collagen. Gels were incubated with MMP activity testing buffer for a gelatin zymography assay and then stained with Coomassie blue (left). Total proteins harvested from whole cell lysis run on 10% SDS were used as internal controls (right)

Quantitative proteomic results and Targetscan prediction suggested that overexpression of miR-133a in myofibroblasts strongly perturbs the transcriptome, particularly myofibroblast markers. Indeed, quantitative RT-PCR analysis indicated that treatment with TGF-β1 for 48 h significantly increased mRNA expression levels of Col1a1 and α-SMA (ACTA2) (Fig. 5a). Cells were further treated with TGF-β1 for an additional 48 h in the presence of miR-133a mimic or control mimic. Compared to control mimic, miR-133a mimic markedly reduced mRNA expression levels of Col1a1 and α-SMA by 77 and 72%, respectively, which is much lower than that of cells prior to this additional 48 h treatment with TGF-β1 (Fig. 5a). Immunostaining of α-SMA also showed a decrease of stress fiber formation after miR-133a overexpression in myofibroblasts (Fig. 5b).

ECM protein deposition in the lung is the primary cause of death of pulmonary fibrosis patients^20^. Matrix metalloproteinase 2 and 9 (MMP-2 and MMP-9) are secreted proteins involved in the breakdown of ECM proteins. Quantitative RT-PCR analysis showed that MMP-2 but not MMP-9 was induced by TGF-β1 treatment, which was not affected by overexpression of miR-133a (Fig. 5c). Gelatin zymography confirmed the unchanged activities of MMP-2 and MMP-9 in cells transfected with miR-133a mimic as compared to cells with control mimic (Fig. 5d). Thus, overexpression of miR-133a reduces the expression of ECM proteins without effects on expression and activity of matrix metalloproteinases.

### MiR-133a directly targets multiple components of TGF-β1 signaling pathways

**Fig. 6:**
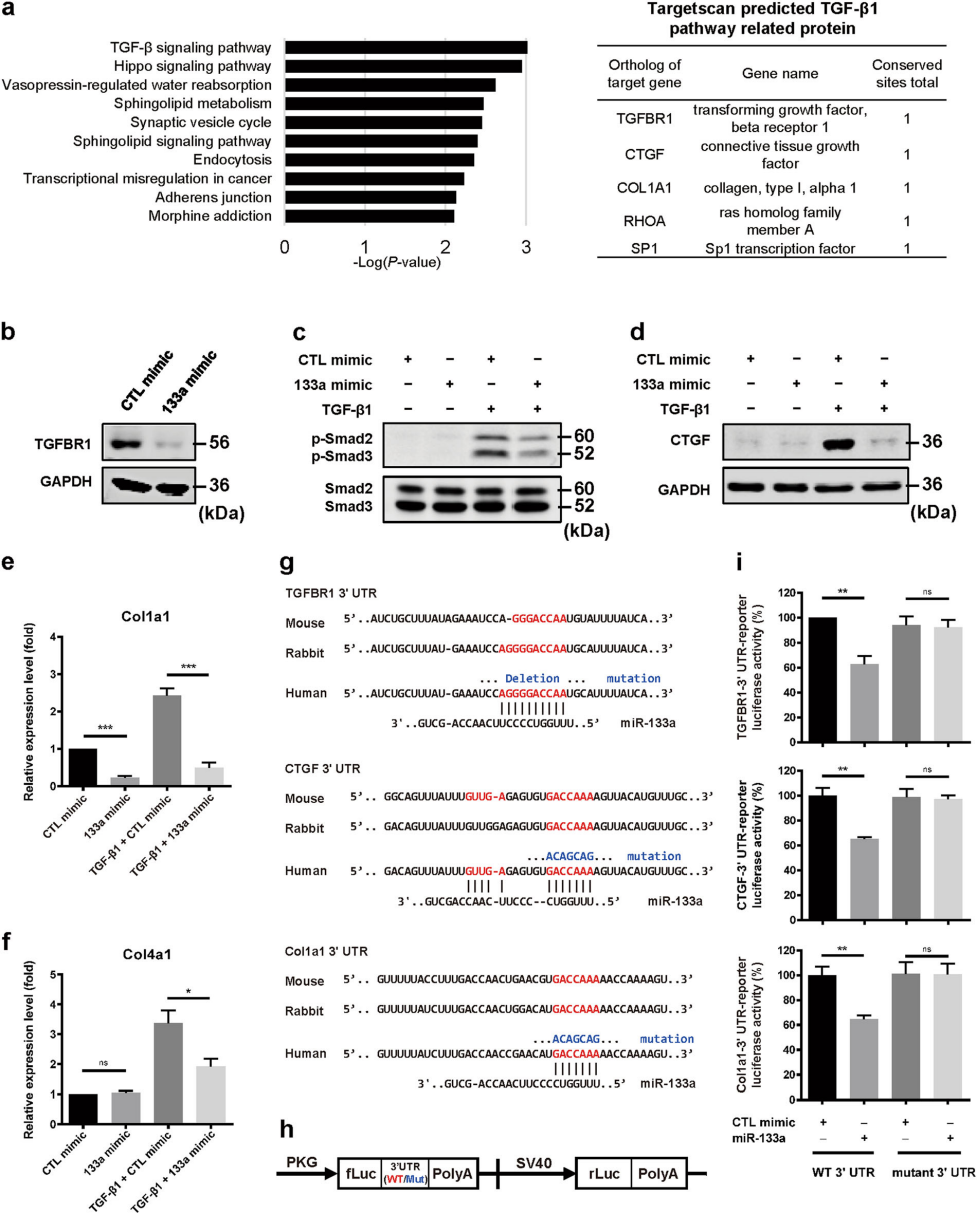
MiR-133a directly targets multiple components of TGF-β1 signaling pathways. **a** KEGG analysis of Targetscan predicted miR-133a targets. HFL cells were transfected with miR-133a mimic or control mimic (CTL) for 24 h, and then stimulated without or with 1 ng/mL TGF-β1 for 48 h. Cells were harvested and subjected to western blot (**b**–**d**) or quantitative RT-PCR analysis (**e**, **f**). Transfection of miR-133a down-regulated TGFBR1 protein expression (**b**), blocked TGF-β1-stimulated Smad2/3 phosphorylation (**c**), CTGF protein expression (**d**), Col1a1 (**e**), and Col4a1 (**f**) mRNA expression. Data are mean ± SEM (*n* = 3–4) with *P *<* 0.05; ***P < 0.001. **g** The 3′UTR of TGFBR1, CTGF or Col1a1 contains a putative miR-133a binding site that is conserved among different species (in red). miR-133a binding site mutations were created by deletion or nucleotide changes (in blue). **h** The 3′UTR fragments containing the putative miR-133a binding site (3’UTR-133a) or their mutants (3′UTR-133aM) of human TGFBR1 (inserted fragment: 1989–2403; seeding region: 2161–2167), CTGF (inserted fragment: 886–1065; seeding region: 1027–1033), or Col1a1 (inserted fragment 78–301; seeding region: 194–200) were subcloned into the pmirGLO reporter vector. **i** These luciferase reporter constructs were transfected into HEK293 cells, along with control or miR-133a mimic. Cells were harvested for firefly luciferase (fLuc) assays with Renilla luciferase (rLuc) as an internal control. Data are mean ± SEM (*n* = 3–4) with **P < 0.01

A total of 605 putative target genes were identified by using miRNA Target Searcher (http://www.targetscan.org/) (Supplementary Table 1), well-established target prediction algorithm for miRNAs. Further KEGG pathway analysis revealed several components of TGF-β signaling pathways such as TGFBR1, CTGF, and Col1A1 as putative miR-133a targets **(**Fig. 6a). Indeed, miR-133a mimic remarkably down-regulated TGFBR1 protein expression in HFL cells (Fig. 6b). Consequently, TGF-β1-induced phosphorylation of Smad2/3 was inhibited by miR-133a mimic (Fig. 6c). Similarly, overexpression of miR-133a also resulted in a significant decrease in TGF-β1-induced expression of CTGF protein and Col1a1 mRNA, respectively (Fig. 6d, e). Interestingly, TGF-β1-induced expression of Col4a1, not a predicted target of miR-133a, was also significantly reduced by miR-133a (Fig. 6f).

We further examined alterations of TGF-β1 signaling pathways by miR-1, a member of the miR-1/miR-133a cluster^21^ that was also upregulated in TGF-β1-treated HFL cells (Table 1 and Fig. 1a). As shown in Supplementary Fig. 3, compared to control mimic, transfection of miR-1 mimic did not change TGFBR1 protein expression or TGF-β1-induced upregulation of CTGF protein or Col1a1 and Col4a1 mRNAs. This is consistent with data showing no effects of miR-1 on TGF-β1-induced α-SMA expression (Fig. 1b). Thus, miR-133a but not miR-1 specifically targets TGF-β1 profibrogenic signaling pathways to inhibit pulmonary fibroblast differentiation.

Alignment of the 3’UTR of TGFBR1, CTGF and Col1a1 among a wide range of species (mouse, rabbit and human) using the Targetscan bioinformatic tool revealed that the predicted binding sites for miR-133a are highly conserved during evolution (Fig. 6g). To determine whether TGFBR1, CTGF and Col1a1 are direct targets of miR-133a, we inserted the fragment of TGFBR1 3′UTR, CTGF 3′UTR or Col1a1 3′UTR containing the miR-133a putative target site (UTR-133a) or their mutants at the seeding region (UTR-133a/M) into the pmirGLO dual-luciferase reporter vector (Fig. 6h). Compared to control mimic, miR-133a mimic reduced the luciferase activity by about 35% (**P < 0.01), which was completely abolished by mutations at their seeding regions (Fig. 6i). These data suggest a novel function of TGF-β1-induced miR-133a as a negative feedback regulator of TGF-β1 profibrogenic signaling pathways in pulmonary fibroblasts via directly repressing TGFBR1, CTGF and collagen expression.

### MiR-133a gene transferred into lung tissues ameliorates bleomycin-induced pulmonary fibrosis in mice

**Fig. 7:**
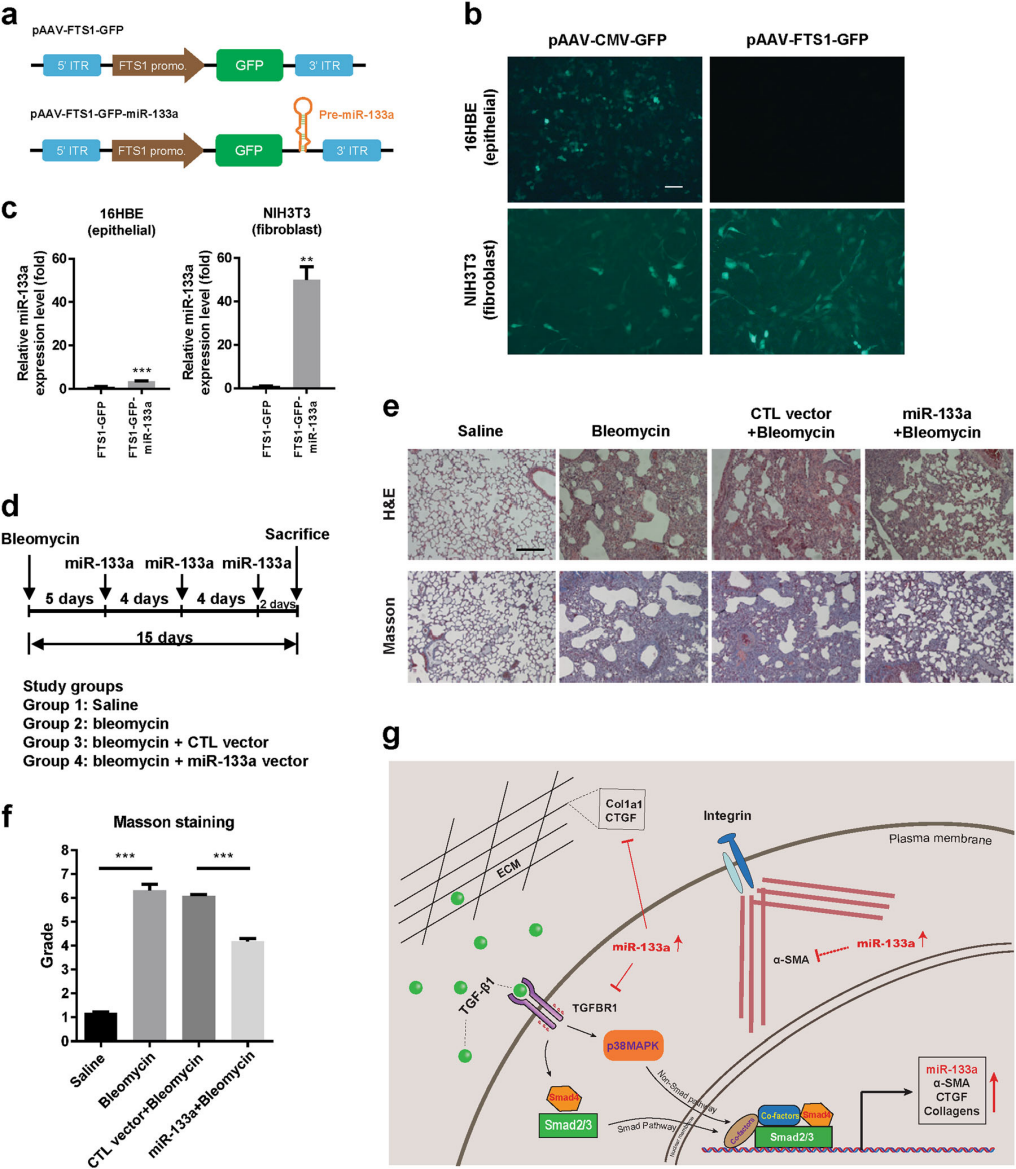
In vivo administration of miR-133a ameliorates experimental pulmonary fibrosis in mice. **a** The cis-element of miR-133a expression vector driven by the FTS1 promoter. **b** Confocal laser scanning microscopy images of GFP expressed in NIH3T3 fibroblasts or epithelial 16HBE cells using pAAV-CMV-GFP or pAAV-FTS1-GFP vector. Scale bar: 50 μm. **c** pAAV-CMV-GFP-133a or pAAV-FTS1-GFP-133a vector was transfected into NIH3T3 fibroblasts or epithelial 16HBE cells. 24 h later, the expression levels of miR-133a were determined by quantitative RT-PCR. **d** Experimental scheme of the mouse model of bleomycin-induced pulmonary fibrosis. Mice were intratracheally injected with saline or bleomycin (50 mg/kg) at day 0. On day 5, mice were administrated pAAV-FTS1-GFP-miR-133a expression vector or its control vector by tail vein injection. This treatment was repeated every 4 days and mice were killed on day 15. **e** Photomicrographs showing H&E staining and Masson staining of lung tissues of mice treated with saline, bleomycin without or with miR-133a expression vector or its control vector. Scale bar: 200 μm. **f** Standardized quantification of pulmonary fibrosis in Masson staining samples by the modified Ashcroft method (score range 0–8). Data are mean ± SEM, *n* = 4, ***P *<* 0.001. **g** Graphical summary of results. TGF-β1-induced miR-133a functions as a feed-back negative regulator of TGF-β1 profibrogenic pathways. TGF-β1 induces miR-133a expression via Smad3 and p38MAPK signaling pathways. Upregulated miR-133a in turn negatively regulates myofibroblast differentiation via targeting multiple components of the TGF-β1 signal pathway such as TGFBR1, CTGF, and Col1a1

The mouse model of bleomycin-induced lung fibrosis is widely used to explore both the pathogenesis of IPF and potential new therapies^22^. We used this mouse model to investigate whether overexpression of miR-133a in vivo has therapeutic benefits. We first constructed a miR-133a expression plasmid pAAV-FTS1-GFP-miR-133a and its control pAAV-FTS1-GFP vector that contains the fibroblast-specific protein-1 (*FSP1*) gene promoter (Fig. 7a). In vitro transfection of this pAAV-FTS1-GFP plasmid into 16HBE epithelial cells and NIH3T3 fibroblast cells showed selective expression of GFP in fibroblasts. In contrast, the plasmid pAAV-CMV-GFP with the CMV promoter had no such selectivity (Fig. 7b). Quantitative RT-PCR confirmed the selective expression of miR-133a in fibroblast cells by the pAAV-FTS1-GFP-133a plasmid (Fig. 7c). Mice were intratracheally administered bleomycin on day 0. After 5 days, the pAAV-FTS1-GFP-miR-133a or pAAV-FTS1-GFP control vector complexed with the Entranster^TM^-in vivo agent was delivered into mice via tail vein injection and this procedure was repeated every 4 days. On day 15, mouse lung tissues were collected and subjected to histochemical analysis (Fig. 7d). H&E and trichrome staining indicated that bleomycin exposure induced collagen deposition in lungs of mice and that administration of the miR-133a vector, but not the control vector, ameliorated bleomycin-induced fibrotic lung lesions in mice (Fig. 7e). Scoring the bleomycin-induced histological fibrosis by the Ashcroft method confirmed pulmonary fibrosis in bleomycin-treated mice as compared with the sham group (6.3 ± 0.3 vs. 1.2 ± 0.1, ***P < 0.001) and administration of the vector expressing miR-133a significantly reduced the Ashcroft score of bleomycin-treated mice from 6.1 ± 0.1 to 4.2 ± 0.1 (Fig. 7f, ***P < 0.001).

## Discussion

Pulmonary fibrosis is characterized by increased pulmonary myofibroblasts with lung stiffening due to an accumulation of extracellular matrix and loss of alveolar cells^23^. Cytokine TGF-β has long been recognized as a major driver of lung fibrosis by promoting differentiation of fibroblasts into myofibroblasts. Surprisingly, our current study identified TGF-β1-induced miR-133a as an anti-fibrotic factor. It functions as a negative feedback regulator of profibrogenic pathways by targeting a panel of TGF-β1 signaling pathway related proteins in human pulmonary fibroblasts.

MicroRNAs are now recognized as important regulators of cellular function in both health and disease^9,24,25^, and are capable of exerting effects both in the cells in which they are synthesized and in other cells via uptake of circulating miRNAs secreted into exosomes^26^. Since TGF-β plays a central role in pulmonary fibrosis by promoting differentiation of fibroblast cells into myofibroblast cells, we performed RNA-sequencing to identify differentially expressed miRNAs in HFL cells with or without TGF-β1 treatment. Our initial hypothesis was that miRNAs upregulated by TGF-β1 may mediate TGF-β1 profibrogenic signaling to promote pulmonary fibrosis. Considering time, cost and other factors, we chose the miR-1/miR-133a cluster for further investigation since it has not been reported to be related to pulmonary fibrosis. We also chose the miR-143/miR-145 cluster and miR-21 as positive controls since these miRNAs were among the TGF-β1-induced miRNAs that promote myofibroblast differentiation and fibrosis^14,27^. Among these five selected miRNAs, transfection of miR-143 or miR-145 enhanced TGF-β1-induced α-SMA expression. Surprisingly, transfection of miR-133a, but not miR-1, another member of the miR-1/miR-133a cluster, inhibited TGF-β1-induced α-SMA expression, suggesting that miR-133a may specifically function as an anti-fibrotic factor.

MiR-133a is among the better studied microRNAs and has been implicated in cancer development, skeletal muscle cell differentiation, as well as in cardiac fibrosis^21,28–30^. Several studies have implicated dysregulation of miR-133a in lung diseases. For example, decreases in miR-133a have been linked to increases in RhoA pathway activity in bronchiolar smooth muscle and airway hyperresponsiveness due to IL-13 or IL-17 treatment^31,32^. However, miR-133a clearly has a broader and more nuanced role in the lungs. We recently identified that increase in miR-133a in airway epithelial cells, whether triggered by exposure to TGF-β or cigarette smoke, is a crucial factor in their epithelial-to-mesenchymal transition in association with increased airway reactivity to methacholine^33^.

The present study has identified an additional role of miR-133a in human pulmonary fibroblasts whose differentiation are regulated by miR-133a. Our data show that in a pre-defined set of miRNAs relevant to pulmonary pathology, miR-133a levels are elevated in differentiated myofibroblasts induced by TGF-β1, and correlate with markers of pulmonary fibrosis. This upregulation of miR-133a by TGF-β1 is time and concentration dependent and was not replicated by another cytokine TNF-α also released by cells during wound healing. TGF-β1-induced miR-133a upregulation was attenuated by inhibitors of Smad3 and p38-MAPK signaling pathways, suggesting that miR-133a may serve as a feedback mediator down-regulating profibrotic genes and desensitizing TGF-β1 signaling pathways. Indeed, TGF-β1 induced expression of α-SMA in these fibroblasts was inhibited by miR-133a mimic while α-SMA expression was enhanced by addition of miR-133a inhibitor. Quantitative proteomics analysis indicated that miR-133a inhibits ECM synthesis and cell motility-related genes in TGF-β1-treated fibroblasts. These findings are in accordance with our RT-PCR and western blot data showing that miR-133a down-regulates the RNAs/proteins of Col1a1, Col4a1 and α-SMA in TGF-β1-treated cells. Interestingly, miR-133a does not alter TGF-β1-induced MMP-2 or MMP-9 gene expression or enzyme activity, thereby creating microenvironment conditions conducive to promoting the degradation of extracellular matrix.

MiRNAs selectively cleavage or bind to mRNAs to affect their stability or translational activity^24,34^. We used another bioinformatic tool (www.targetscan.org) to identify possible targets of miR-133a. KEGG analysis of the Targetscan similarly predicted a down-regulation of ECM-related profibrotic genes such as CTGF, collagens, fibronectin, and suggested that the TGF-β signaling pathway is a prominent target. In line with our analysis, Col1a1 was recently identified as a direct target of miR-133a in a different context^35^. Thus, both predicted direct targets CTGF and Col1a1 or un-predicted targets Col4a1 and α-SMA are down-regulated after miR-133a overexpression, strongly suggesting an anti-fibrotic role of miR-133a in pulmonary fibrosis.

Furthermore, miR-133a treatment of human pulmonary myofibroblasts also upregulates oxidative phosphorylation and the citric acid cycle, which are consistent with miR-133a inducing the formation of a more quiescent fibroblast phenotype (i.e., fibrocyte). Thus, miR-133a treated myofibroblasts have a slenderer spindle shape without α-SMA expression, show decreases in focal adhesion markers and extracellular matrix protein synthesis, and have likely switched to efficient aerobic Krebs cycle metabolism to generate energy rather than utilizing anaerobic glycolysis for cell growth and proliferation. Our observations suggesting miR-133a-induced alterations in cellular metabolism are supported by the findings of impaired exercise tolerance, mitochondrial biogenesis, and skeletal muscle fiber structure and maintenance in miR-133a-deficient mice^36,37^.

Currently, median survival time for IPF patients is only 2–3 years from diagnosis and limited effective therapies for IPF exist. Thus, there is an unmet need for novel anti-fibrotic therapies. MiRNA-based therapeutic approaches may represent a promising alternative to current approaches in treatment of IPF^38,39^. The combined data obtained from cultured human pulmonary fibroblasts pre-exposed to TGF-β1 showed that they reverted to a smaller resting phenotype with diminished synthesis of contractile and extracellular matrix proteins following miR-133a treatment. These in vitro findings predicted that selective expression of miR-133a in pulmonary fibroblasts may limit or perhaps even reverse pulmonary fibrosis in vivo. Therefore, we generated a novel fibroblast-targeted miR-133a expression vector and tested our prediction using a mouse model of bleomycin-induced pulmonary fibrosis. Five days after administering bleomycin to mice to induce experimental pulmonary fibrosis, this vector was administered intravenously. Three miR-133a vector treatments caused an easily discernible and significant decrease in bleomycin-induced lung fibrosis and preservation of normal lung structure 10 days later. These data confirm that selective upregulation of miR-133a in pulmonary fibroblasts in vivo exerts therapeutically beneficial anti-fibrotic effects. Although additional work is required to optimize this miRNA-based strategy to inhibit or even reverse pulmonary fibrosis, modulating the expression of miR-133a in fibroblasts may provide a new therapeutic target for pulmonary fibrosis treatment. Further investigation of whether miR-133a deficiency (knockout) exacerbates bleomycin-induced pulmonary fibrosis in mice or in vivo gain of miR-133a (knock-in) protects mice from bleomycin-induced pulmonary fibrosis will provide direct evidence and increase the validity of miR-133a as therapeutics against pulmonary fibrosis.

In conclusion, our study shows that an increase in intracellular miR-133a exerts anti-fibrotic effects on pulmonary fibroblasts. This molecular mediator alters intracellular protein expression in fibroblasts that had been driven by TGF-β1 into the contractile myofibroblast phenotype. Multiple myofibroblast proteins and pathways are targeted during this process with levels of some key proteins being decreased while others are increased. Proteins directly decreased by miR-133a include TGF-β1 receptor, CTGF and collagen that are directly involved in the ability of these cells to transform, proliferate and secrete extracellular matrix in response to TGF-β1 (Fig. 7g). Proteins increased in TGF-β1-stimulated pulmonary fibroblasts by miR-133a include those involved in oxidative phosphorylation. Unchanged by miR-133a upregulation is TGF-β1-stimulated expression of matrix metalloproteases that degrade extracellular matrix. Altogether, this creates an efficiently metabolizing quiescent fibroblast phenotype with reduced extracellular matrix synthesis which should favor removal of extracellular matrix. Anticipated beneficial effects of miR-133a upregulation in pulmonary fibroblasts were found in the bleomycin-induced mouse model of pulmonary fibrosis. These data are an exciting breakthrough given the current dismal prognosis associated with IPF in humans. It should be noted that any therapeutic plan for using miR-133a to treat IPF must include strategies to directly target the fibroblasts due to undesirable off-target effects such as epithelial-to-mesenchymal transitions. Thus, identifying additional fibroblast-selective mechanisms that might increase intracellular miR-133a expression in these cells and potential beneficial/synergistic interactions with other miRNAs that have been associated with IPF should be explored. We are currently collaborating with a medicinal chemist to select the most potent/efficacious compounds that are specific for miR-133a upregulation and action pathways in pulmonary fibroblasts. Of particular interest in pulmonary fibrosis is the fact that these therapies can be selectively delivered to the lung by inhalation. Our long-term goal is to determine if targeting fibroblast miR-133a expression via local drug application provides an effective strategy for prevention and/or treatment of IPF. At a minimum, our study provides legitimate hope that effective therapies for fibrosis-associated morbidity and mortality such as that due to IPF are on the horizon.

## Materials and methods

### Cell culture, reagents, and antibodies

HEK293 cells and mouse fibroblast NIH3T3 cells were obtained from the American Type Culture Collection (ATCC, Manassas, VA, USA). Human primary lung fibroblast cells (HFL) were established by Dr. Reynold Panettieri’s laboratory from patients with brain-related disease but no history of pulmonary fibrosis^40^. The cells were frozen at an early passage and cultured for a maximum of fifteen passages. The cells were maintained at 37 °C with 5% CO_2_ in Dulbecco’s Modified Eagle’s Medium/Ham’s Nutrient Mixture F-12 (DME/F12, Life Technologies/Gibco, Grand Island, NY) supplemented with 10% fetal bovine serum (GE Healthcare Bio-Sciences, Pittsburgh, PA, USA), 100 U/mL penicillin G and 100 μg/mL streptomycin (Life Technologies/Gibco). Smad3 inhibitor SIS3 and p38MAPK inhibitor SB203580 were purchased from (AdooQ BioScience, Irvine, CA, USA). Recombinant human TGF-β1 and the Smad2/3 Antibody Sampler Kit were obtained from Cell Signaling Technology (Danvers, MA, USA). The mouse anti-α-SMA monoclonal antibody was obtained from Sigma-Aldrich (St. Louis, MO, USA). The rabbit anti-TGFBR1 antibody was obtained from Merck Millipore (Billerica, MA, USA). The mouse anti-CTGF monoclonal antibody, mouse anti-GAPDH monoclonal antibody were obtained from Proteintech (Chicago, IL, USA). The GAPDH levels served as internal controls.

### MiRNA sequencing

MiRNA sequencing was performed as previously described^33^. Briefly, total RNA was isolated from samples using mirVana miRNA Isolation Kit (Invitrogen) according to the manufacturer’s instructions. For small RNA sequencing, RNA quality was check by Fragment Analyzer™ Automated CE System; a NEXTflex^TM^ Small RNA-Seq Kit v3 (Bioo Scientific #5132-05) was used with 1 µg of total RNA for the construction of small RNA sequencing libraries according to the standard Illumina protocols; miRNA-sequencing was performed with Illumina Nextseq 500 at the University of Nebraska Medical Center Next Generation Sequencing Core Facility (Omaha, NE, USA). miRNAs of HFLs cells treated with 1 ng/mL TGF-β1 or saline were sequenced. All sequencing data including upregulated and down-regulated miRNAs have been deposited in NCBI’s Gene Expression Omnibus and the accession number is GSE125183. The quantitative miRNA expression analysis was performed using the app (B&Gu @ University of Torino) in BaseSpace Sequence Hub.

### Quantitative RT-PCR

Total RNA was isolated using TRIzol® Reagent (Thermo Fisher Scientific, Waltham, MA). RT-PCR reactions for miRNA detection were determined by TaqMan probe based microRNA assay on total RNA and normalized to RUN6B levels according to a previous report^41^. Quantitative RT-PCR was performed using UltraSYBR Mixture (CWBiotech) and normalized by GAPDH levels. Primers and probes used are listed in Supplementary Table 2. The amplification procedure consisted of 95 °C for 10 min, followed by 40 cycles of 95 °C for 15 s and 60 °C for 60 s. The relative expression levels between samples were calculated using the comparative delta CT (threshold cycle number) method (2^−ΔΔCT^) with a control sample as the reference point.

### Small RNAs and plasmid transfection

MiRNA mimics or inhibitors (mimic negative control: miR01101-1-5, miR-133a mimic: miR10000427-1-5, miR-1 mimic: miR10000416-1-5, miR-143 mimic: miR10000435-1-5, miR-145 mimic: miR10000437-1-5, miR-21 mimic: miR10000076-1-5, inhibitor negative control: miR02101-1-5, miR-133a inhibitor: miR20000427-1-5) were purchased from RiboBio (Guangzhou, China). Small RNA and plasmid transfections were performed with Lipofectamine® RNAiMAX Reagent and Lipofectamine® 3000 Reagent (Thermo Fisher Scientific), respectively, according to the manufacturer’s instructions.

### MiRNA targets prediction

Targetscan algorithms were used to identify the putative miR-133a targets^42^. Annotated 3′-UTRs for upregulated or down-regulated genes in miR-133a overexpressing cells, were scanned for strict miRNA targets (8mer, 7mer-m8, and 7mer-A1). The significance of the measured overlaps was calculated using a hypergeometric test.

### Western blot

HFL cells were lysed in buffer containing 0.2% sodium deoxycholate, 50 mM Tris-HCl, 1% TritonX-100, 15 mM NaCl, 0.1% SDS and 1 × Protease inhibitor cocktail (Sigma) at 4 °C for 20 min. Cell lysates were centrifuged at 12,000 × *g* for 20 min, the supernatant was collected, and protein concentration was determined with a Pierce BCA Kit (Rockford, IL, USA). Samples were then subjected to western blot analysis as reported^43,44^. Briefly, samples were resolved by 10% SDS-PAGE, transferred to Immobilon-FL PVDF membrane (Millipore), and probed with various primary antibodies. Membranes were then incubated with IRDye® 800CW- or 680RD- conjugated secondary antibodies and visualized using a LI-COR Odyssey Imaging System (LI-COR Biosciences, Lincoln, NE, USA).

### Immunofluorescence staining for α-SMA

HFL cell α-SMA was visualized with an anti-α-SMA primary antibody (Sigma) and an Alexa Fluor 488-labeled secondary antibody (Life Technologies). The images were obtained by using Olympus FluoView® FV1200 confocal laser scanning microscope (Olympus Corporation, Center Valley, PA).

### Plasmid construction

To construct luciferase reporter plasmid containing TGF-β receptor 1 (TGFBR1)-3UTR-133a, CTGF-3UTR-133a, or collagen type 1-alpha1 **(**Col1a1)-3UTR-133a, the 3′UTR fragments containing miR-133a binding sites/mutant sites of human TGFBR1, CTGF or Col1a1 were synthesized at Genewiz (Beijing, China) with *Xba*I and *Sac*I cutting site and cloned into the pmirGLO reporter vector (Promega, Fitchburg, WI). miR-133a binding site mutants are shown by the underlined nucleotide changes.

The FTS1 promoter that is selectively activated in fibroblasts^45,46^ was cloned into the pAAV-CMV-GFP plasmid (Cell Biolabs, San Diego, CA, USA) to replace the CMV promoter. The miR-133a with flanking sequence was amplified from human genomic DNAs and then was cloned into the pAAV-CMV-GFP plasmid or pAAV-FTS1-GFP vector to generate pAAV-CMV-GFP-miR-133a or pAAV-FTS1-GFP-miR-133a. Primers and probes used are listed in Supplementary Table 2. All constructs were validated by Sanger sequencing.

### Luciferase reporter assay

In all, 0.5 µg of the pmirGLO luciferase constructs containing the wild-type or mutants of 3′ UTR fragments of TGFBR1, CTGF or Col1a1, were co-transfected with control mimics or miR-133a mimics into HEK-293 cells using Lipofectamine 3000 according to the manufacturer’s instructions. Cells were harvested 24 h later and then subjected to luciferase activity assays using a Dual-Glo® Luciferase Assay kit (Promega) and Sirius luciferase assay system (Berthold, Germany) as we previously reported^33^.

### Gelatin zymography

Gelatin zymography was performed as previously described^47^. Briefly, gels (SDS-PAGE, 10%) were co-polymerized with gelatin (1 mg/mL) (Sigma-Aldrich). Equal amounts of samples were loaded onto the wells of the gel. After electrophoresis, gels were washed in renaturing buffer (2.5% Triton X-100 in 50 mM Tris-HCl, pH 7.5) for 3 h at room temperature and followed by 18 h of incubation at 37 °C in developing buffer (10 mM CaCl_2_, 200 mM NaCl in 50 mM Tris-HCl, pH 7.5). Gels were then stained with Coomassie blue and de-stained with 30% methanol and 10% acetic acid. Gels were photographed by EPSON Perfection V500 (Long Beach, CA, USA).

### Quantitative proteomics and bioinformatics analysis

Cells were lysed by sonication in 8 M urea/0.1 M Tris-HCl (pH 8.0)/1 × Protease Inhibitor Cocktail (Sigma). Cell lysates were reduced with 10 mM DTT for 2 h at room temperature followed by alkylation with 20 mM iodoacetamide for 30 min in the dark. Samples were trypsinized in 50 mM triethylammonium bicarbonate (TEAB) at 37 °C overnight, then desalted and eluted with 60% acetonitrile. Peptides were lyophilized and re-dissolved with 100 mM TEAB buffer. 100 μg of protein of each sample was labeled with TMT six-plex® (Thermo Fisher Scientific) according to the manufacturer’s instructions. Samples were fractionated using an L-3000 HPLC System (Rigol, Beijing, China) and all nanoLC-MS/MS experiments were performed on a Q Exactive equipped with an Easy n-LC 1000 HPLC system (Thermo Fisher Scientific) as previously described^48^.

The raw data from Q Exactive were analyzed with Proteome Discovery version 2.2.0.388 using Sequest HT search engine for protein identification and Percolator for FDR (false discovery rate) analysis. The Uniprot Human protein database was used and FDR < 0.05 was set for protein identification. Normalization to the protein median of each sample was used to correct for experimental bias and the normalization mode was selected as total peptide amount. Among the 5394 total proteins detected, differentially regulated proteins (*P* < 0.05) were identified by multiple ANOVA analysis in conjunction with Dunnet’s analysis using R (3.2) and their associations with signaling pathways in HFL cells were explored using the Kyoto Encyclopedia of Genes and Genomes (KEGG) database resource (www.kegg.jp) and the R package clusterProfiler that automates the process of biological-term classification and the enrichment analysis of gene clusters^49^. Briefly, genes in each pathway for Homo sapiens were extracted from the KEGG database. Fish’s extract test was further applied to identify enriched pathways (unadjusted *P* < 0.05). Differentially expressed proteins were further validated by immunoblotting or RT-PCR.

### Bleomycin-induced mouse pulmonary fibrosis and histologic analysis

Female C57BL/6JCnc mice (6–8 weeks of age) were obtained from Vital River Experimental Animal Center (Beijing, China) and maintained in a specific pathogen-free environment. All experiments were performed according to the guidelines for experimental animals and approved by the Institutional Animal Care and Use Committee of the Institute of Biophysics, Chinese Academy of Sciences.

The mice were intratracheally administered with either bleomycin (50 mg/kg per mouse in 50 μL of saline; Zhejiang Hisun Pharmaceutical Co., Ltd., Taizhou, China) or vehicle (control) on Day 0^22,40^. To overexpress miR-133a specifically in mouse lung fibroblasts, 25 μg of pAAV-FTS1-GFP or pAAV-FTS1-GFP-miR-133a complexed with Entranster^TM^-in vivo (Engreen Biosystem Co, Ltd., Beijing, China) was injected via the tail vein on Day 5 and every 4 days thereafter. The mice (*n* = 4) were killed on Day 15. Lungs were collected and inflated with 4% paraformaldehyde for 2 days and embedded in paraffin before sectioning into 5-μm-thick slices.

Sections of paraformaldehyde-fixed mouse lungs were analyzed by hematoxylin and eosin (H&E) or Masson’s trichrome staining to assess fibrotic changes in the lungs as described^40^. Photos of at least 15 fields from multiple sections of each mouse lung were taken at ×200 magnification and scored separately by the modified Ashcroft method (score range 0–8)^40,50,51^. The pulmonary fibrosis histopathology score of each mouse is expressed as the mean score of at least 15 photos.

### Statistical analysis

All values are expressed as the mean ± SEM of at least three independent experiments. Data were analyzed using the GraphPad Prism software (GraphPad Software, Inc, San Diego, CA, USA). The Student’s unpaired *t* test (two-tailed) was used for comparison between two groups. *P < 0.05 was considered statistically significant; ns, not significant.

## Supplementary information

Supplementary Figure 1.

Supplementary Figure 2.

Supplementary Figure 3.

Supplementary Table 1.

Supplementary Table 2.

Supplementary figure legends.
