# Supplementary figures and images for: Transforming growth factor (TGF)-β1-induced miR-133a inhibits myofibroblast differentiation and pulmonary fibrosis
Source: Cell Death Dis. 2019 Sep 11;10(9):670. doi: 10.1038/s41419-019-1873-x (PMC6739313; doi:10.1038/s41419-019-1873-x)

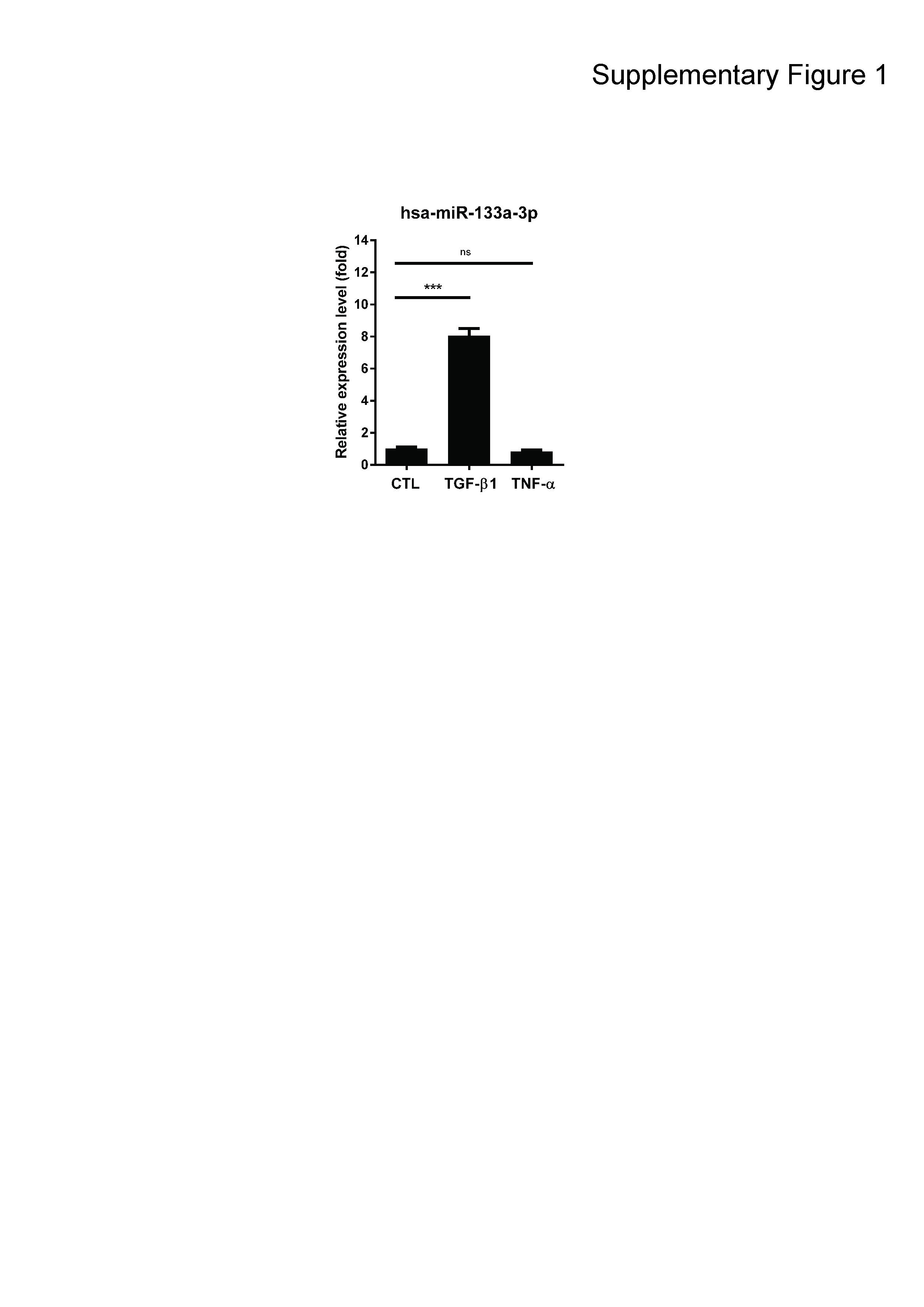

Supplement: Supplementary file 1 — Supplementary Figure 1. [file 41419_2019_1873_MOESM1_ESM.tif]

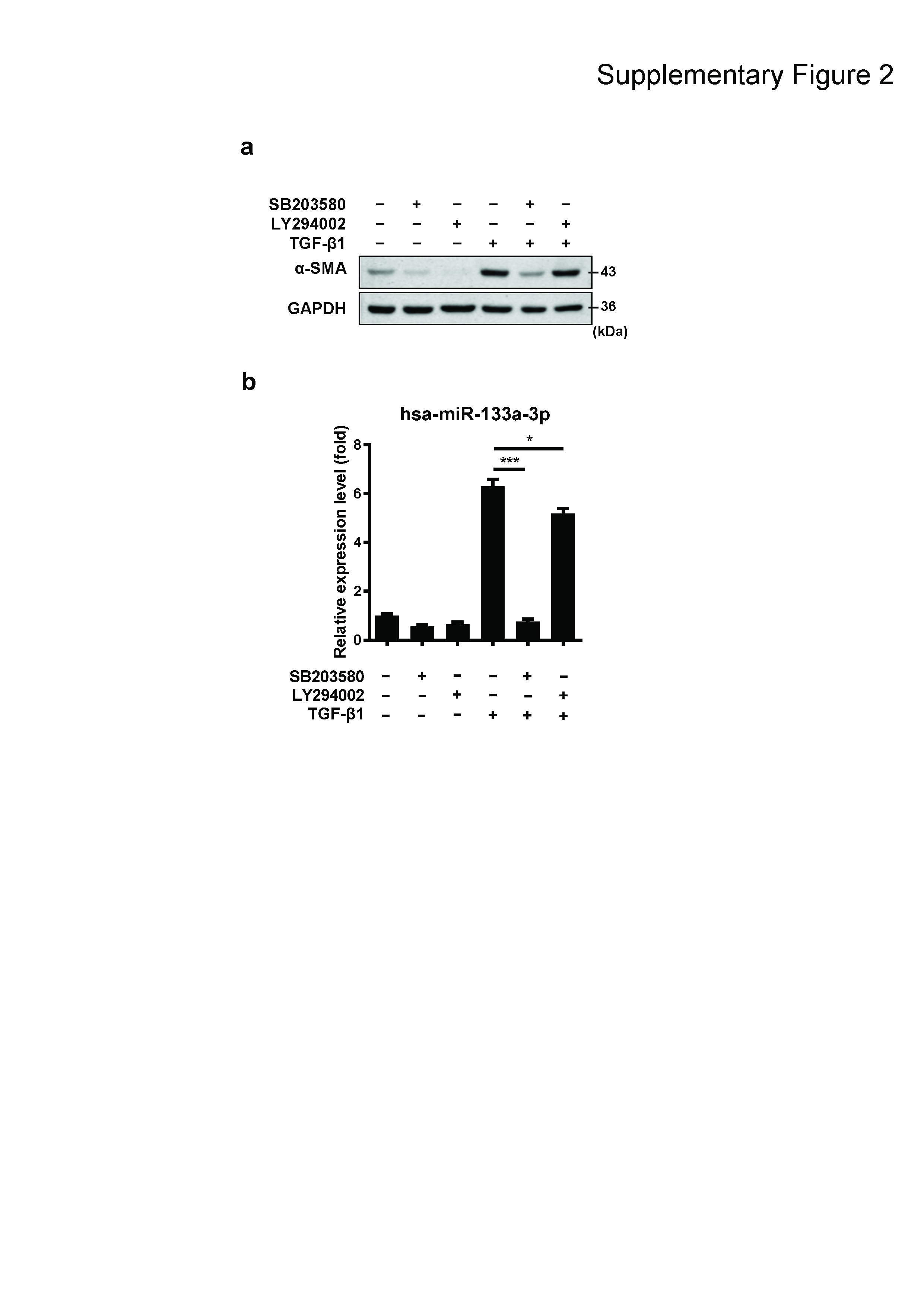

Supplement: Supplementary file 2 — Supplementary Figure 2. [file 41419_2019_1873_MOESM2_ESM.tif]

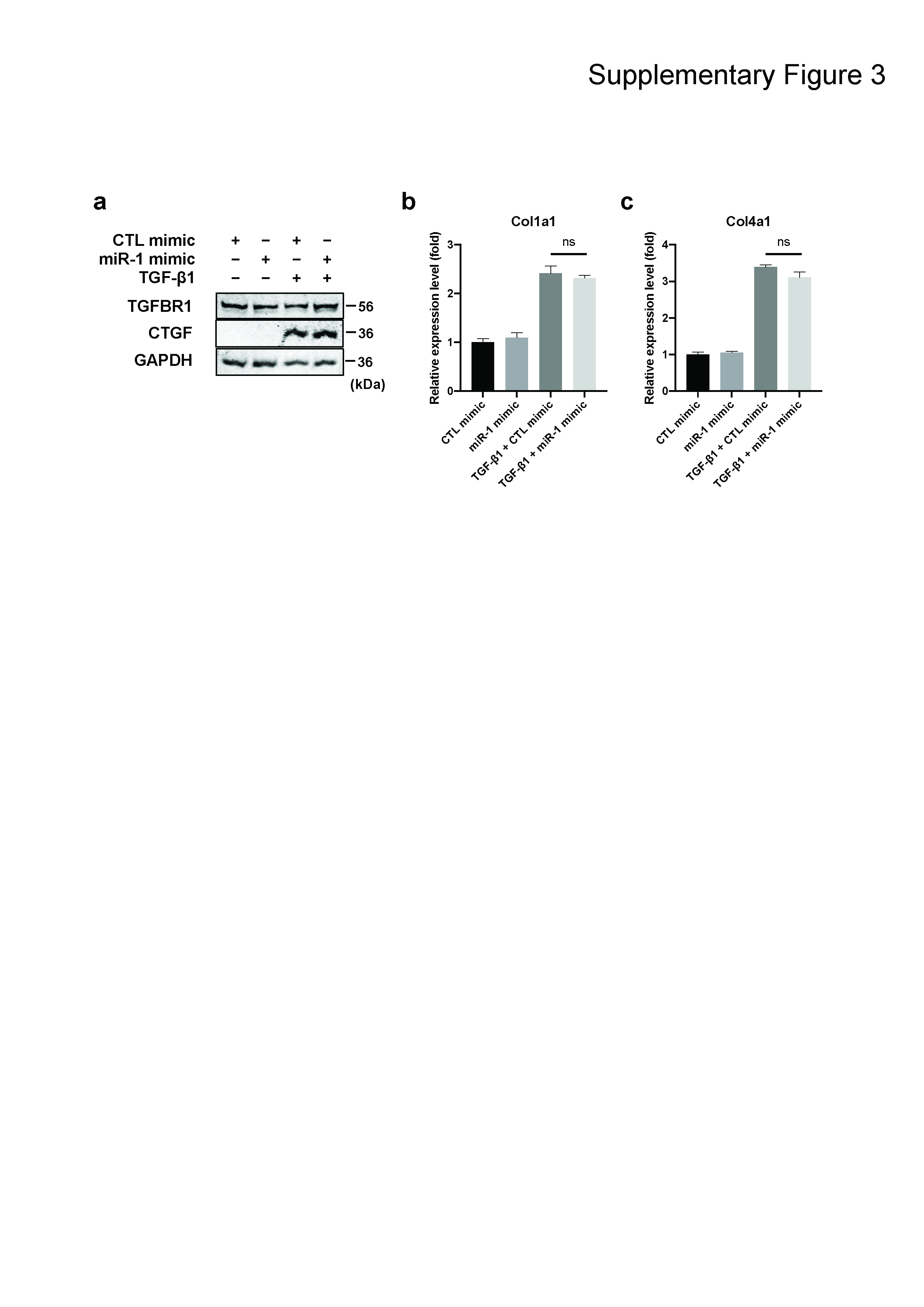

Supplement: Supplementary file 3 — Supplementary Figure 3. [file 41419_2019_1873_MOESM3_ESM.tif]
