# Supplementary material for: Transforming growth factor (TGF)-β1-induced miR-133a inhibits myofibroblast differentiation and pulmonary fibrosis
Source: Cell Death Dis. 2019 Sep 11;10(9):670. doi: 10.1038/s41419-019-1873-x (PMC6739313; doi:10.1038/s41419-019-1873-x)
Supplement: Supplementary file 4 — Supplementary Table 1. [file 41419_2019_1873_MOESM4_ESM.docx]

| TargetScan7.2 miR-133a-3p predicted targets | | |
| --- | --- | --- |
| Target gene | Representative transcript | Gene name |
| LHFP | ENST00000379589.3 | lipoma HMGIC fusion partner |
| SEC61B | ENST00000498603.1 | Sec61 beta subunit |
| TAGLN2 | ENST00000368096.1 | transgelin 2 |
| CETN3 | ENST00000283122.3 | centrin, EF-hand protein, 3 |
| FTL | ENST00000331825.6 | ferritin, light polypeptide |
| LDLRAP1 | ENST00000374338.4 | low density lipoprotein receptor adaptor protein 1 |
| PTBP1 | ENST00000350092.4 | polypyrimidine tract binding protein 1 |
| CLTA | ENST00000433436.2 | clathrin, light chain A |
| SLC50A1 | ENST00000368404.4 | solute carrier family 50 (sugar efflux transporter), member 1 |
| SYT2 | ENST00000367267.1 | synaptotagmin II |
| TMEM200B | ENST00000521452.1 | transmembrane protein 200B |
| TIMM17A | ENST00000367287.4 | translocase of inner mitochondrial membrane 17 homolog A (yeast) |
| BICC1 | ENST00000373886.3 | bicaudal C homolog 1 (Drosophila) |
| VKORC1 | ENST00000300851.6 | vitamin K epoxide reductase complex, subunit 1 |
| ZNF354A | ENST00000335815.2 | zinc finger protein 354A |
| SAMD5 | ENST00000367474.1 | sterile alpha motif domain containing 5 |
| LASP1 | ENST00000318008.6 | LIM and SH3 protein 1 |
| PPP2CA | ENST00000481195.1 | protein phosphatase 2, catalytic subunit, alpha isozyme |
| PAX7 | ENST00000420770.2 | paired box 7 |
| C12orf43 | ENST00000536407.2 | chromosome 12 open reading frame 43 |
| PPP2CB | ENST00000221138.4 | protein phosphatase 2, catalytic subunit, beta isozyme |
| SLC30A7 | ENST00000370112.4 | solute carrier family 30 (zinc transporter), member 7 |
| SGMS2 | ENST00000394684.4 | sphingomyelin synthase 2 |
| GDNF | ENST00000326524.2 | glial cell derived neurotrophic factor |
| SUMO1 | ENST00000392246.2 | small ubiquitin-like modifier 1 |
| FOSL2 | ENST00000379619.1 | FOS-like antigen 2 |
| SIMC1 | ENST00000443967.1 | SUMO-interacting motifs containing 1 |
| SMARCD1 | ENST00000394963.4 | SWI/SNF related, matrix associated, actin dependent regulator of chromatin, subfamily d, member 1 |
| GPM6A | ENST00000280187.7 | glycoprotein M6A |
| DOLPP1 | ENST00000540102.1 | dolichyldiphosphatase 1 |
| PTPRZ1 | ENST00000393386.2 | protein tyrosine phosphatase, receptor-type, Z polypeptide 1 |
| TMEM167A | ENST00000502346.1 | transmembrane protein 167A |
| CMPK1 | ENST00000371873.5 | cytidine monophosphate (UMP-CMP) kinase 1, cytosolic |
| PPP2R2D | ENST00000422256.2 | protein phosphatase 2, regulatory subunit B, delta |
| LHX9 | ENST00000367390.3 | LIM homeobox 9 |
| KIRREL | ENST00000368172.1 | kin of IRRE like (Drosophila) |
| XXYLT1 | ENST00000310380.6 | xyloside xylosyltransferase 1 |
| RBMX | ENST00000562646.1 | RNA binding motif protein, X-linked |
| VPS54 | ENST00000409558.4 | vacuolar protein sorting 54 homolog (S. cerevisiae) |
| COL25A1 | ENST00000399132.1 | collagen, type XXV, alpha 1 |
| AGRP | ENST00000290953.2 | agouti related protein homolog (mouse) |
| SGPP1 | ENST00000247225.6 | sphingosine-1-phosphate phosphatase 1 |
| FOXL2 | ENST00000330315.3 | forkhead box L2 |
| ZNF131 | ENST00000505606.2 | zinc finger protein 131 |
| KIF3C | ENST00000264712.3 | kinesin family member 3C |
| EMP2 | ENST00000359543.3 | epithelial membrane protein 2 |
| EIF4A1 | ENST00000582746.1 | eukaryotic translation initiation factor 4A1 |
| NRIP3 | ENST00000309166.3 | nuclear receptor interacting protein 3 |
| AL117190.3 | ENST00000599197.1 | Esophagus cancer-related gene-2 interaction susceptibility protein; Uncharacterized protein |
| B3GALNT1 | ENST00000320474.4 | beta-1,3-N-acetylgalactosaminyltransferase 1 (globoside blood group) |
| RAP2C | ENST00000342983.2 | RAP2C, member of RAS oncogene family |
| GABPB2 | ENST00000368918.3 | GA binding protein transcription factor, beta subunit 2 |
| ELFN1 | ENST00000424383.2 | extracellular leucine-rich repeat and fibronectin type III domain containing 1 |
| TXLNA | ENST00000373610.3 | taxilin alpha |
| STOM | ENST00000286713.2 | stomatin |
| ANKRD28 | ENST00000399451.2 | ankyrin repeat domain 28 |
| SLC6A6 | ENST00000454876.2 | solute carrier family 6 (neurotransmitter transporter), member 6 |
| ZC3H11A | ENST00000332127.4 | zinc finger CCCH-type containing 11A |
| SFXN2 | ENST00000369893.5 | sideroflexin 2 |
| DCLRE1A | ENST00000361384.2 | DNA cross-link repair 1A |
| PFAS | ENST00000314666.6 | phosphoribosylformylglycinamidine synthase |
| TTPAL | ENST00000372906.2 | tocopherol (alpha) transfer protein-like |
| KIAA1429 | ENST00000437199.1 | KIAA1429 |
| QKI | ENST00000392127.2 | QKI, KH domain containing, RNA binding |
| GBA2 | ENST00000378088.1 | glucosidase, beta (bile acid) 2 |
| SF3B4 | ENST00000271628.8 | splicing factor 3b, subunit 4, 49kDa |
| TMEM170B | ENST00000379426.1 | transmembrane protein 170B |
| TSPAN18 | ENST00000340160.3 | tetraspanin 18 |
| GCH1 | ENST00000491895.2 | GTP cyclohydrolase 1 |
| RBMXL1 | ENST00000321792.5 | RNA binding motif protein, X-linked-like 1 |
| DCBLD1 | ENST00000338728.5 | discoidin, CUB and LCCL domain containing 1 |
| SH3GL2 | ENST00000380607.4 | SH3-domain GRB2-like 2 |
| ZNF280C | ENST00000370978.4 | zinc finger protein 280C |
| SLC39A1 | ENST00000356205.4 | solute carrier family 39 (zinc transporter), member 1 |
| VAMP2 | ENST00000404970.3 | vesicle-associated membrane protein 2 (synaptobrevin 2) |
| METTL21B | ENST00000333012.5 | methyltransferase like 21B |
| FOXC1 | ENST00000380874.2 | forkhead box C1 |
| PTPRK | ENST00000368226.4 | protein tyrosine phosphatase, receptor type, K |
| WASF2 | ENST00000536657.1 | WAS protein family, member 2 |
| RAVER1 | ENST00000293677.6 | ribonucleoprotein, PTB-binding 1 |
| TRAM2 | ENST00000182527.3 | translocation associated membrane protein 2 |
| SEPHS2 | ENST00000542752.1 | selenophosphate synthetase 2 |
| SOBP | ENST00000317357.5 | sine oculis binding protein homolog (Drosophila) |
| FAM46A | ENST00000369754.3 | family with sequence similarity 46, member A |
| MAP3K2 | ENST00000409947.1 | mitogen-activated protein kinase kinase kinase 2 |
| TM9SF3 | ENST00000371142.4 | transmembrane 9 superfamily member 3 |
| RARB | ENST00000437042.2 | retinoic acid receptor, beta |
| SPRN | ENST00000414069.2 | shadow of prion protein homolog (zebrafish) |
| ZC3H7A | ENST00000396516.2 | zinc finger CCCH-type containing 7A |
| JDP2 | ENST00000435893.2 | Jun dimerization protein 2 |
| POU6F2 | ENST00000518318.2 | POU class 6 homeobox 2 |
| SNX15 | ENST00000377244.3 | sorting nexin 15 |
| SNRK | ENST00000429705.2 | SNF related kinase |
| GARNL3 | ENST00000314904.5 | GTPase activating Rap/RanGAP domain-like 3 |
| RAD51L3-RFFL | ENST00000593039.1 | Uncharacterized protein |
| CELF6 | ENST00000287202.5 | CUGBP, Elav-like family member 6 |
| LHX5 | ENST00000261731.3 | LIM homeobox 5 |
| HS2ST1 | ENST00000370550.5 | heparan sulfate 2-O-sulfotransferase 1 |
| SGK1 | ENST00000367858.5 | serum/glucocorticoid regulated kinase 1 |
| ATP6AP2 | ENST00000378438.4 | ATPase, H+ transporting, lysosomal accessory protein 2 |
| ELF3 | ENST00000367284.5 | E74-like factor 3 (ets domain transcription factor, epithelial-specific ) |
| NUP160 | ENST00000378460.2 | nucleoporin 160kDa |
| ANKRD46 | ENST00000335659.3 | ankyrin repeat domain 46 |
| CERS2 | ENST00000368954.5 | ceramide synthase 2 |
| ENPP5 | ENST00000371383.2 | ectonucleotide pyrophosphatase/phosphodiesterase 5 (putative) |
| CECR6 | ENST00000399875.1 | cat eye syndrome chromosome region, candidate 6 |
| PDE1C | ENST00000396193.1 | phosphodiesterase 1C, calmodulin-dependent 70kDa |
| SV2A | ENST00000369146.3 | synaptic vesicle glycoprotein 2A |
| DUSP1 | ENST00000239223.3 | dual specificity phosphatase 1 |
| HAPLN1 | ENST00000274341.4 | hyaluronan and proteoglycan link protein 1 |
| THRAP3 | ENST00000354618.5 | thyroid hormone receptor associated protein 3 |
| VAT1 | ENST00000355653.3 | vesicle amine transport 1 |
| NDRG1 | ENST00000323851.7 | N-myc downstream regulated 1 |
| SYT1 | ENST00000457153.2 | synaptotagmin I |
| FGF1 | ENST00000360966.5 | fibroblast growth factor 1 (acidic) |
| SP3 | ENST00000310015.6 | Sp3 transcription factor |
| CRK | ENST00000398970.5 | v-crk avian sarcoma virus CT10 oncogene homolog |
| YPEL2 | ENST00000312655.4 | yippee-like 2 (Drosophila) |
| ANKRD44 | ENST00000282272.8 | ankyrin repeat domain 44 |
| MECOM | ENST00000460814.1 | MDS1 and EVI1 complex locus |
| MED12L | ENST00000474524.1 | mediator complex subunit 12-like |
| RIMS1 | ENST00000348717.5 | regulating synaptic membrane exocytosis 1 |
| SCN2B | ENST00000278947.5 | sodium channel, voltage-gated, type II, beta subunit |
| PFKFB3 | ENST00000536985.1 | 6-phosphofructo-2-kinase/fructose-2,6-biphosphatase 3 |
| ARFIP2 | ENST00000254584.2 | ADP-ribosylation factor interacting protein 2 |
| BCORL1 | ENST00000540052.1 | BCL6 corepressor-like 1 |
| ATOX1 | ENST00000521264.1 | antioxidant 1 copper chaperone |
| RFFL | ENST00000315249.7 | ring finger and FYVE-like domain containing E3 ubiquitin protein ligase |
| SACM1L | ENST00000389061.5 | SAC1 suppressor of actin mutations 1-like (yeast) |
| PFN2 | ENST00000239940.7 | profilin 2 |
| CLCN6 | ENST00000312413.6 | chloride channel, voltage-sensitive 6 |
| FGFR1 | ENST00000397091.5 | fibroblast growth factor receptor 1 |
| UBE2Q1 | ENST00000292211.4 | ubiquitin-conjugating enzyme E2Q family member 1 |
| BTBD10 | ENST00000278174.5 | BTB (POZ) domain containing 10 |
| CCDC176 | ENST00000394009.3 | coiled-coil domain containing 176 |
| PLCL2 | ENST00000418129.2 | phospholipase C-like 2 |
| FOXP4 | ENST00000373063.3 | forkhead box P4 |
| GZF1 | ENST00000338121.5 | GDNF-inducible zinc finger protein 1 |
| XPO4 | ENST00000400602.2 | exportin 4 |
| SNX30 | ENST00000374232.3 | sorting nexin family member 30 |
| CORO1C | ENST00000261401.3 | coronin, actin binding protein, 1C |
| LOXL4 | ENST00000260702.3 | lysyl oxidase-like 4 |
| RPL17-C18orf32 | ENST00000584895.1 | RPL17-C18orf32 readthrough |
| RB1CC1 | ENST00000025008.5 | RB1-inducible coiled-coil 1 |
| EXD2 | ENST00000409018.3 | exonuclease 3'-5' domain containing 2 |
| SHISA5 | ENST00000296444.2 | shisa family member 5 |
| YES1 | ENST00000577961.1 | v-yes-1 Yamaguchi sarcoma viral oncogene homolog 1 |
| GXYLT1 | ENST00000398675.3 | glucoside xylosyltransferase 1 |
| FBXL2 | ENST00000484457.1 | F-box and leucine-rich repeat protein 2 |
| SYNRG | ENST00000339208.6 | synergin, gamma |
| ARHGAP12 | ENST00000311380.4 | Rho GTPase activating protein 12 |
| RBM23 | ENST00000555209.1 | RNA binding motif protein 23 |
| VEGFC | ENST00000280193.2 | vascular endothelial growth factor C |
| TNFRSF10B | ENST00000276431.4 | tumor necrosis factor receptor superfamily, member 10b |
| CAP1 | ENST00000372797.3 | CAP, adenylate cyclase-associated protein 1 (yeast) |
| CTBP2 | ENST00000337195.5 | C-terminal binding protein 2 |
| RAPH1 | ENST00000319170.5 | Ras association (RalGDS/AF-6) and pleckstrin homology domains 1 |
| MEIS2 | ENST00000397624.3 | Meis homeobox 2 |
| FAM117B | ENST00000392238.2 | family with sequence similarity 117, member B |
| NAA40 | ENST00000377793.4 | N(alpha)-acetyltransferase 40, NatD catalytic subunit |
| SLMO2 | ENST00000355937.4 | slowmo homolog 2 (Drosophila) |
| PML | ENST00000565898.1 | promyelocytic leukemia |
| VAPB | ENST00000395802.3 | VAMP (vesicle-associated membrane protein)-associated protein B and C |
| SOGA3 | ENST00000556132.1 | SOGA family member 3 |
| ELP5 | ENST00000574993.1 | elongator acetyltransferase complex subunit 5 |
| LAMB3 | ENST00000356082.4 | laminin, beta 3 |
| LPIN2 | ENST00000261596.4 | lipin 2 |
| CEP85L | ENST00000368491.3 | centrosomal protein 85kDa-like |
| AKAP9 | ENST00000356239.3 | A kinase (PRKA) anchor protein 9 |
| PPFIA2 | ENST00000549396.1 | protein tyrosine phosphatase, receptor type, f polypeptide (PTPRF), interacting protein (liprin), alpha 2 |
| CELF1 | ENST00000395290.2 | CUGBP, Elav-like family member 1 |
| SOCS2 | ENST00000548537.1 | suppressor of cytokine signaling 2 |
| ETF1 | ENST00000499810.2 | eukaryotic translation termination factor 1 |
| ELAVL1 | ENST00000407627.2 | ELAV like RNA binding protein 1 |
| SGTB | ENST00000381007.4 | small glutamine-rich tetratricopeptide repeat (TPR)-containing, beta |
| RCE1 | ENST00000309657.3 | RCE1 homolog, prenyl protein protease (S. cerevisiae) |
| DAPK2 | ENST00000261891.3 | death-associated protein kinase 2 |
| PTBP2 | ENST00000609116.1 | polypyrimidine tract binding protein 2 |
| KIAA1432 | ENST00000414202.2 | KIAA1432 |
| RFC1 | ENST00000349703.2 | replication factor C (activator 1) 1, 145kDa |
| PLEKHA8 | ENST00000449726.1 | pleckstrin homology domain containing, family A (phosphoinositide binding specific) member 8 |
| TGFB2 | ENST00000366930.4 | transforming growth factor, beta 2 |
| CMTM6 | ENST00000205636.3 | CKLF-like MARVEL transmembrane domain containing 6 |
| UBXN7 | ENST00000296328.4 | UBX domain protein 7 |
| PTPN22 | ENST00000460620.1 | protein tyrosine phosphatase, non-receptor type 22 (lymphoid) |
| FBXW11 | ENST00000296933.6 | F-box and WD repeat domain containing 11 |
| PAOX | ENST00000368539.4 | polyamine oxidase (exo-N4-amino) |
| LRRC7 | ENST00000310961.5 | leucine rich repeat containing 7 |
| ZNF740 | ENST00000416904.3 | zinc finger protein 740 |
| KCNA6 | ENST00000433855.1 | potassium voltage-gated channel, shaker-related subfamily, member 6 |
| MSN | ENST00000360270.5 | moesin |
| ARPC5 | ENST00000359856.6 | actin related protein 2/3 complex, subunit 5, 16kDa |
| DSN1 | ENST00000426836.1 | DSN1, MIS12 kinetochore complex component |
| SLC25A36 | ENST00000446041.2 | solute carrier family 25 (pyrimidine nucleotide carrier ), member 36 |
| ARL3 | ENST00000260746.5 | ADP-ribosylation factor-like 3 |
| XXbac-BPG32J3.20 | ENST00000461287.1 |  |
| STX5 | ENST00000377897.4 | syntaxin 5 |
| RAB30 | ENST00000533486.1 | RAB30, member RAS oncogene family |
| CTGF | ENST00000367976.3 | connective tissue growth factor |
| PTPRD | ENST00000381196.4 | protein tyrosine phosphatase, receptor type, D |
| LDOC1 | ENST00000370526.2 | leucine zipper, down-regulated in cancer 1 |
| RBPJ | ENST00000504907.1 | recombination signal binding protein for immunoglobulin kappa J region |
| PRDM6 | ENST00000407847.4 | PR domain containing 6 |
| PPFIA3 | ENST00000334186.4 | protein tyrosine phosphatase, receptor type, f polypeptide (PTPRF), interacting protein (liprin), alpha 3 |
| C20orf194 | ENST00000453730.2 | chromosome 20 open reading frame 194 |
| RBMS1 | ENST00000348849.3 | RNA binding motif, single stranded interacting protein 1 |
| KCND3 | ENST00000369697.1 | potassium voltage-gated channel, Shal-related subfamily, member 3 |
| SUPT16H | ENST00000216297.2 | suppressor of Ty 16 homolog (S. cerevisiae) |
| CTSV | ENST00000259470.5 | cathepsin V |
| KCTD20 | ENST00000373731.2 | potassium channel tetramerization domain containing 20 |
| MLLT3 | ENST00000380338.4 | myeloid/lymphoid or mixed-lineage leukemia (trithorax homolog, Drosophila); translocated to, 3 |
| PRDM1 | ENST00000369089.3 | PR domain containing 1, with ZNF domain |
| CDK8 | ENST00000536792.1 | cyclin-dependent kinase 8 |
| PITPNM2 | ENST00000280562.5 | phosphatidylinositol transfer protein, membrane-associated 2 |
| TEAD1 | ENST00000361905.4 | TEA domain family member 1 (SV40 transcriptional enhancer factor) |
| RUNX1T1 | ENST00000523629.1 | runt-related transcription factor 1; translocated to, 1 (cyclin D-related) |
| PAPD5 | ENST00000357464.3 | PAP associated domain containing 5 |
| SLC7A8 | ENST00000469263.1 | solute carrier family 7 (amino acid transporter light chain, L system), member 8 |
| TCF7 | ENST00000518915.1 | transcription factor 7 (T-cell specific, HMG-box) |
| AOX1 | ENST00000374700.2 | aldehyde oxidase 1 |
| ELF2 | ENST00000394235.2 | E74-like factor 2 (ets domain transcription factor) |
| GRM5 | ENST00000418177.2 | glutamate receptor, metabotropic 5 |
| EPHA7 | ENST00000369303.4 | EPH receptor A7 |
| STXBP6 | ENST00000396700.1 | syntaxin binding protein 6 (amisyn) |
| DCP1A | ENST00000607628.1 | decapping mRNA 1A |
| THNSL1 | ENST00000524413.1 | threonine synthase-like 1 (S. cerevisiae) |
| ST8SIA3 | ENST00000324000.3 | ST8 alpha-N-acetyl-neuraminide alpha-2,8-sialyltransferase 3 |
| TMEM57 | ENST00000399766.3 | transmembrane protein 57 |
| TOR2A | ENST00000458505.3 | torsin family 2, member A |
| PEX5L | ENST00000467460.1 | peroxisomal biogenesis factor 5-like |
| SLC35F5 | ENST00000245680.2 | solute carrier family 35, member F5 |
| PITPNB | ENST00000335272.5 | phosphatidylinositol transfer protein, beta |
| ARHGAP9 | ENST00000550288.1 | Rho GTPase activating protein 9 |
| GMEB1 | ENST00000373816.1 | glucocorticoid modulatory element binding protein 1 |
| PREX1 | ENST00000396220.1 | phosphatidylinositol-3,4,5-trisphosphate-dependent Rac exchange factor 1 |
| SVOP | ENST00000299134.5 | SV2 related protein homolog (rat) |
| KIAA0430 | ENST00000396368.3 | KIAA0430 |
| C1QL1 | ENST00000253407.3 | complement component 1, q subcomponent-like 1 |
| PSEN1 | ENST00000344094.3 | presenilin 1 |
| BCL2L1 | ENST00000376062.2 | BCL2-like 1 |
| GDI2 | ENST00000380191.4 | GDP dissociation inhibitor 2 |
| PURB | ENST00000395699.2 | purine-rich element binding protein B |
| SLC25A39 | ENST00000225308.8 | solute carrier family 25, member 39 |
| IL6ST | ENST00000381287.4 | interleukin 6 signal transducer (gp130, oncostatin M receptor) |
| EFNA4 | ENST00000368409.3 | ephrin-A4 |
| MYH9 | ENST00000216181.5 | myosin, heavy chain 9, non-muscle |
| TENM1 | ENST00000371130.3 | teneurin transmembrane protein 1 |
| SLC4A1 | ENST00000262418.6 | solute carrier family 4 (anion exchanger), member 1 |
| CNKSR2 | ENST00000379510.3 | connector enhancer of kinase suppressor of Ras 2 |
| NIPA2 | ENST00000337451.3 | non imprinted in Prader-Willi/Angelman syndrome 2 |
| DNAJB1 | ENST00000254322.2 | DnaJ (Hsp40) homolog, subfamily B, member 1 |
| RWDD2A | ENST00000369724.4 | RWD domain containing 2A |
| FAM49A | ENST00000381323.3 | family with sequence similarity 49, member A |
| ENC1 | ENST00000302351.4 | ectodermal-neural cortex 1 (with BTB domain) |
| KLHL42 | ENST00000381271.2 | kelch-like family member 42 |
| MTMR4 | ENST00000579925.1 | myotubularin related protein 4 |
| FAM43B | ENST00000332947.4 | family with sequence similarity 43, member B |
| TK2 | ENST00000299697.7 | thymidine kinase 2, mitochondrial |
| B3GNT9 | ENST00000449549.3 | UDP-GlcNAc:betaGal beta-1,3-N-acetylglucosaminyltransferase 9 |
| SOGA1 | ENST00000279034.6 | suppressor of glucose, autophagy associated 1 |
| DYNC1LI2 | ENST00000258198.2 | dynein, cytoplasmic 1, light intermediate chain 2 |
| GPR173 | ENST00000332582.4 | G protein-coupled receptor 173 |
| FLVCR1 | ENST00000366971.4 | feline leukemia virus subgroup C cellular receptor 1 |
| SCOC | ENST00000608372.1 | short coiled-coil protein |
| KPNA6 | ENST00000373625.3 | karyopherin alpha 6 (importin alpha 7) |
| TPD52 | ENST00000379096.5 | tumor protein D52 |
| FAM193B | ENST00000514747.1 | family with sequence similarity 193, member B |
| TMEM158 | ENST00000503771.1 | transmembrane protein 158 (gene/pseudogene) |
| ADRA2B | ENST00000409345.3 | adrenoceptor alpha 2B |
| AP1B1 | ENST00000357586.2 | adaptor-related protein complex 1, beta 1 subunit |
| POLH | ENST00000372226.1 | polymerase (DNA directed), eta |
| MAML3 | ENST00000509479.2 | mastermind-like 3 (Drosophila) |
| CELF5 | ENST00000541430.2 | CUGBP, Elav-like family member 5 |
| CKAP4 | ENST00000378026.4 | cytoskeleton-associated protein 4 |
| ZC3HAV1 | ENST00000242351.5 | zinc finger CCCH-type, antiviral 1 |
| IDH1 | ENST00000345146.2 | isocitrate dehydrogenase 1 (NADP+), soluble |
| CDIP1 | ENST00000564828.1 | cell death-inducing p53 target 1 |
| AFAP1 | ENST00000360265.4 | actin filament associated protein 1 |
| KLHL9 | ENST00000359039.4 | kelch-like family member 9 |
| SLC41A1 | ENST00000367137.3 | solute carrier family 41 (magnesium transporter), member 1 |
| ERMP1 | ENST00000381506.3 | endoplasmic reticulum metallopeptidase 1 |
| GDI1 | ENST00000447750.2 | GDP dissociation inhibitor 1 |
| DLGAP3 | ENST00000373347.1 | discs, large (Drosophila) homolog-associated protein 3 |
| GCLC | ENST00000229416.6 | glutamate-cysteine ligase, catalytic subunit |
| DIAPH2 | ENST00000324765.8 | diaphanous-related formin 2 |
| CREB5 | ENST00000357727.2 | cAMP responsive element binding protein 5 |
| CACUL1 | ENST00000369151.3 | CDK2-associated, cullin domain 1 |
| EEF1A1 | ENST00000316292.9 | eukaryotic translation elongation factor 1 alpha 1 |
| TFE3 | ENST00000315869.7 | transcription factor binding to IGHM enhancer 3 |
| WNT4 | ENST00000290167.6 | wingless-type MMTV integration site family, member 4 |
| LZTS2 | ENST00000370223.3 | leucine zipper, putative tumor suppressor 2 |
| SLC8A2 | ENST00000236877.6 | solute carrier family 8 (sodium/calcium exchanger), member 2 |
| GRM7 | ENST00000486284.1 | glutamate receptor, metabotropic 7 |
| LETMD1 | ENST00000380123.2 | LETM1 domain containing 1 |
| CCDC144A | ENST00000443444.2 | coiled-coil domain containing 144A |
| ZNF436 | ENST00000314011.4 | zinc finger protein 436 |
| SESN1 | ENST00000436639.2 | sestrin 1 |
| FRS2 | ENST00000550389.1 | fibroblast growth factor receptor substrate 2 |
| SLC7A2 | ENST00000494857.1 | solute carrier family 7 (cationic amino acid transporter, y+ system), member 2 |
| MAP3K3 | ENST00000361357.3 | mitogen-activated protein kinase kinase kinase 3 |
| TMEM110 | ENST00000355083.5 | transmembrane protein 110 |
| UNC13A | ENST00000519716.2 | unc-13 homolog A (C. elegans) |
| TTC31 | ENST00000410003.1 | tetratricopeptide repeat domain 31 |
| KLHDC10 | ENST00000335420.5 | kelch domain containing 10 |
| FSCN1 | ENST00000382361.3 | fascin homolog 1, actin-bundling protein (Strongylocentrotus purpuratus) |
| HS3ST5 | ENST00000312719.5 | heparan sulfate (glucosamine) 3-O-sulfotransferase 5 |
| AP2M1 | ENST00000382456.3 | adaptor-related protein complex 2, mu 1 subunit |
| MMP14 | ENST00000311852.6 | matrix metallopeptidase 14 (membrane-inserted) |
| KLF7 | ENST00000423015.1 | Kruppel-like factor 7 (ubiquitous) |
| ANKRD52 | ENST00000267116.7 | ankyrin repeat domain 52 |
| FAM160B1 | ENST00000369248.4 | family with sequence similarity 160, member B1 |
| ASH1L | ENST00000368346.3 | ash1 (absent, small, or homeotic)-like (Drosophila) |
| FOXG1 | ENST00000382535.3 | forkhead box G1 |
| ABCD2 | ENST00000308666.3 | ATP-binding cassette, sub-family D (ALD), member 2 |
| TGFBR1 | ENST00000374994.4 | transforming growth factor, beta receptor 1 |
| SLC30A5 | ENST00000396591.3 | solute carrier family 30 (zinc transporter), member 5 |
| ZBTB14 | ENST00000357006.4 | zinc finger and BTB domain containing 14 |
| POU4F1 | ENST00000377208.5 | POU class 4 homeobox 1 |
| SMIM14 | ENST00000295958.5 | small integral membrane protein 14 |
| CDK5R1 | ENST00000313401.3 | cyclin-dependent kinase 5, regulatory subunit 1 (p35) |
| KLHL11 | ENST00000319121.3 | kelch-like family member 11 |
| COL1A1 | ENST00000225964.5 | collagen, type I, alpha 1 |
| MLXIP | ENST00000319080.7 | MLX interacting protein |
| FAM19A5 | ENST00000358295.5 | family with sequence similarity 19 (chemokine (C-C motif)-like), member A5 |
| PDIK1L | ENST00000374271.4 | PDLIM1 interacting kinase 1 like |
| CPLX2 | ENST00000359546.4 | complexin 2 |
| DLG2 | ENST00000398309.2 | discs, large homolog 2 (Drosophila) |
| MRFAP1 | ENST00000382581.4 | Morf4 family associated protein 1 |
| PTBP3 | ENST00000374257.1 | polypyrimidine tract binding protein 3 |
| MAN1A2 | ENST00000356554.3 | mannosidase, alpha, class 1A, member 2 |
| MEIS1 | ENST00000488550.1 | Meis homeobox 1 |
| KLHL2 | ENST00000538127.1 | kelch-like family member 2 |
| HOXD1 | ENST00000331462.4 | homeobox D1 |
| NCKAP5L | ENST00000335999.6 | NCK-associated protein 5-like |
| IFIT2 | ENST00000371826.3 | interferon-induced protein with tetratricopeptide repeats 2 |
| PIK3C2A | ENST00000265970.7 | phosphatidylinositol-4-phosphate 3-kinase, catalytic subunit type 2 alpha |
| ORAI2 | ENST00000356387.2 | ORAI calcium release-activated calcium modulator 2 |
| PRDM16 | ENST00000511072.1 | PR domain containing 16 |
| UBFD1 | ENST00000395878.3 | ubiquitin family domain containing 1 |
| CCDC30 | ENST00000342022.4 | coiled-coil domain containing 30 |
| ADAMTS5 | ENST00000284987.5 | ADAM metallopeptidase with thrombospondin type 1 motif, 5 |
| HSPA13 | ENST00000285667.3 | heat shock protein 70kDa family, member 13 |
| EYA1 | ENST00000388742.4 | eyes absent homolog 1 (Drosophila) |
| USP6 | ENST00000332776.4 | ubiquitin specific peptidase 6 (Tre-2 oncogene) |
| CSNK1G3 | ENST00000360683.2 | casein kinase 1, gamma 3 |
| NTNG1 | ENST00000370067.1 | netrin G1 |
| CDYL2 | ENST00000570137.2 | chromodomain protein, Y-like 2 |
| SLC25A51 | ENST00000380590.3 | solute carrier family 25, member 51 |
| ZNF395 | ENST00000344423.5 | zinc finger protein 395 |
| NCAM2 | ENST00000400546.1 | neural cell adhesion molecule 2 |
| CDCA8 | ENST00000373055.1 | cell division cycle associated 8 |
| PPP6R3 | ENST00000393800.2 | protein phosphatase 6, regulatory subunit 3 |
| NELFA | ENST00000382882.3 | negative elongation factor complex member A |
| ZHX1 | ENST00000395571.3 | zinc fingers and homeoboxes 1 |
| ITPRIPL2 | ENST00000381440.3 | inositol 1,4,5-trisphosphate receptor interacting protein-like 2 |
| USH1G | ENST00000319642.1 | Usher syndrome 1G (autosomal recessive) |
| FAM13A | ENST00000395002.2 | family with sequence similarity 13, member A |
| ARX | ENST00000379044.4 | aristaless related homeobox |
| NELL2 | ENST00000395487.2 | NEL-like 2 (chicken) |
| NEBL | ENST00000377122.4 | nebulette |
| KCNJ12 | ENST00000583088.1 | potassium inwardly-rectifying channel, subfamily J, member 12 |
| ADCY6 | ENST00000357869.3 | adenylate cyclase 6 |
| WTAP | ENST00000358372.4 | Wilms tumor 1 associated protein |
| OTUD7B | ENST00000369135.4 | OTU domain containing 7B |
| BAZ2A | ENST00000379441.3 | bromodomain adjacent to zinc finger domain, 2A |
| ELMO1 | ENST00000341056.3 | engulfment and cell motility 1 |
| EPS15 | ENST00000371730.2 | epidermal growth factor receptor pathway substrate 15 |
| ESYT1 | ENST00000394048.5 | extended synaptotagmin-like protein 1 |
| PPP2R4 | ENST00000393370.2 | protein phosphatase 2A activator, regulatory subunit 4 |
| WDR59 | ENST00000262144.6 | WD repeat domain 59 |
| PARD6G | ENST00000353265.3 | par-6 family cell polarity regulator gamma |
| RFX3 | ENST00000382004.3 | regulatory factor X, 3 (influences HLA class II expression) |
| LIN7A | ENST00000552864.1 | lin-7 homolog A (C. elegans) |
| KDM2A | ENST00000398645.2 | lysine (K)-specific demethylase 2A |
| DOT1L | ENST00000398665.3 | DOT1-like histone H3K79 methyltransferase |
| PCDH17 | ENST00000377918.3 | protocadherin 17 |
| PPP2R5E | ENST00000337537.3 | protein phosphatase 2, regulatory subunit B', epsilon isoform |
| NR6A1 | ENST00000487099.2 | nuclear receptor subfamily 6, group A, member 1 |
| PDE10A | ENST00000366882.1 | phosphodiesterase 10A |
| RHOA | ENST00000454011.2 | ras homolog family member A |
| MAFG | ENST00000357736.4 | v-maf avian musculoaponeurotic fibrosarcoma oncogene homolog G |
| MRGBP | ENST00000370487.3 | MRG/MORF4L binding protein |
| PSD3 | ENST00000327040.8 | pleckstrin and Sec7 domain containing 3 |
| XPO1 | ENST00000401558.2 | exportin 1 (CRM1 homolog, yeast) |
| KCNK7 | ENST00000394216.2 | potassium channel, subfamily K, member 7 |
| SFT2D3 | ENST00000310981.4 | SFT2 domain containing 3 |
| C17orf96 | ENST00000325814.5 | chromosome 17 open reading frame 96 |
| FOXP2 | ENST00000408937.3 | forkhead box P2 |
| AK2 | ENST00000467905.1 | adenylate kinase 2 |
| DBNL | ENST00000494774.1 | drebrin-like |
| LIN7C | ENST00000278193.2 | lin-7 homolog C (C. elegans) |
| PTMA | ENST00000409115.3 | prothymosin, alpha |
| DSTYK | ENST00000367160.4 | dual serine/threonine and tyrosine protein kinase |
| MTX3 | ENST00000509852.1 | metaxin 3 |
| PCSK6 | ENST00000348070.1 | proprotein convertase subtilisin/kexin type 6 |
| NRG3 | ENST00000372142.2 | neuregulin 3 |
| ZBTB4 | ENST00000380599.4 | zinc finger and BTB domain containing 4 |
| SMPD4 | ENST00000351288.6 | sphingomyelin phosphodiesterase 4, neutral membrane (neutral sphingomyelinase-3) |
| SCN1A | ENST00000423058.2 | sodium channel, voltage-gated, type I, alpha subunit |
| MMGT1 | ENST00000305963.2 | membrane magnesium transporter 1 |
| CD47 | ENST00000361309.5 | CD47 molecule |
| EYA4 | ENST00000367895.5 | eyes absent homolog 4 (Drosophila) |
| SLC4A8 | ENST00000453097.2 | solute carrier family 4, sodium bicarbonate cotransporter, member 8 |
| GALNT16 | ENST00000337827.4 | UDP-N-acetyl-alpha-D-galactosamine:polypeptide N-acetylgalactosaminyltransferase 16 |
| LRRC2 | ENST00000395905.3 | leucine rich repeat containing 2 |
| PDK3 | ENST00000441463.2 | pyruvate dehydrogenase kinase, isozyme 3 |
| KIAA0232 | ENST00000425103.1 | KIAA0232 |
| AMD1 | ENST00000368885.3 | adenosylmethionine decarboxylase 1 |
| PCSK5 | ENST00000376752.4 | proprotein convertase subtilisin/kexin type 5 |
| LTBP1 | ENST00000404525.1 | latent transforming growth factor beta binding protein 1 |
| AIF1L | ENST00000372300.1 | allograft inflammatory factor 1-like |
| SFXN5 | ENST00000410065.1 | sideroflexin 5 |
| DDX3Y | ENST00000336079.3 | DEAD (Asp-Glu-Ala-Asp) box helicase 3, Y-linked |
| GABBR2 | ENST00000259455.2 | gamma-aminobutyric acid (GABA) B receptor, 2 |
| KIF21A | ENST00000361961.3 | kinesin family member 21A |
| SIX5 | ENST00000560168.1 | SIX homeobox 5 |
| PEAK1 | ENST00000312493.4 | pseudopodium-enriched atypical kinase 1 |
| SMC2 | ENST00000374793.3 | structural maintenance of chromosomes 2 |
| PHTF2 | ENST00000416283.2 | putative homeodomain transcription factor 2 |
| HIVEP2 | ENST00000367604.1 | human immunodeficiency virus type I enhancer binding protein 2 |
| SYNPO2L | ENST00000372873.4 | synaptopodin 2-like |
| ESRRG | ENST00000361525.3 | estrogen-related receptor gamma |
| ZNF217 | ENST00000371471.2 | zinc finger protein 217 |
| HLTF | ENST00000465259.1 | helicase-like transcription factor |
| ZBTB38 | ENST00000514251.1 | zinc finger and BTB domain containing 38 |
| ARHGAP39 | ENST00000377307.2 | Rho GTPase activating protein 39 |
| EPHB4 | ENST00000360620.3 | EPH receptor B4 |
| HOXB5 | ENST00000239151.5 | homeobox B5 |
| LPGAT1 | ENST00000366997.4 | lysophosphatidylglycerol acyltransferase 1 |
| JAZF1 | ENST00000283928.5 | JAZF zinc finger 1 |
| ZNF362 | ENST00000539719.1 | zinc finger protein 362 |
| DNAJC3 | ENST00000602402.1 | DnaJ (Hsp40) homolog, subfamily C, member 3 |
| ZBTB10 | ENST00000430430.1 | zinc finger and BTB domain containing 10 |
| ZNF664 | ENST00000538932.2 | zinc finger protein 664 |
| MYO9B | ENST00000595618.1 | myosin IXB |
| INSR | ENST00000341500.5 | insulin receptor |
| E2F7 | ENST00000416496.2 | E2F transcription factor 7 |
| C2orf69 | ENST00000319974.5 | chromosome 2 open reading frame 69 |
| KIAA1522 | ENST00000401073.2 | KIAA1522 |
| NFYA | ENST00000341376.6 | nuclear transcription factor Y, alpha |
| SLC33A1 | ENST00000392845.3 | solute carrier family 33 (acetyl-CoA transporter), member 1 |
| SMARCA2 | ENST00000349721.2 | SWI/SNF related, matrix associated, actin dependent regulator of chromatin, subfamily a, member 2 |
| CSRNP1 | ENST00000273153.5 | cysteine-serine-rich nuclear protein 1 |
| FAM199X | ENST00000493442.1 | family with sequence similarity 199, X-linked |
| NUS1 | ENST00000368494.3 | nuclear undecaprenyl pyrophosphate synthase 1 homolog (S. cerevisiae) |
| SPRY4 | ENST00000344120.4 | sprouty homolog 4 (Drosophila) |
| DUSP7 | ENST00000495880.1 | dual specificity phosphatase 7 |
| GNB4 | ENST00000232564.3 | guanine nucleotide binding protein (G protein), beta polypeptide 4 |
| ACTR1A | ENST00000487599.1 | ARP1 actin-related protein 1 homolog A, centractin alpha (yeast) |
| UBE2Z | ENST00000360943.5 | ubiquitin-conjugating enzyme E2Z |
| THUMPD3 | ENST00000345094.3 | THUMP domain containing 3 |
| RFX6 | ENST00000332958.2 | regulatory factor X, 6 |
| SPTSSA | ENST00000298130.4 | serine palmitoyltransferase, small subunit A |
| TBL1X | ENST00000407597.2 | transducin (beta)-like 1X-linked |
| ALDH5A1 | ENST00000357578.3 | aldehyde dehydrogenase 5 family, member A1 |
| TET3 | ENST00000409262.3 | tet methylcytosine dioxygenase 3 |
| CNNM2 | ENST00000369878.4 | cyclin M2 |
| VPS13B | ENST00000395996.1 | vacuolar protein sorting 13 homolog B (yeast) |
| SETD6 | ENST00000394266.4 | SET domain containing 6 |
| ZNRF3 | ENST00000544604.2 | zinc and ring finger 3 |
| FBN1 | ENST00000316623.5 | fibrillin 1 |
| SGSM1 | ENST00000400358.4 | small G protein signaling modulator 1 |
| LZTS3 | ENST00000329152.3 | Homo sapiens leucine zipper, putative tumor suppressor family member 3 (LZTS3), transcript variant 2, mRNA. |
| MIER3 | ENST00000381226.3 | mesoderm induction early response 1, family member 3 |
| CNOT1 | ENST00000317147.5 | CCR4-NOT transcription complex, subunit 1 |
| ANAPC1 | ENST00000341068.3 | anaphase promoting complex subunit 1 |
| UNC80 | ENST00000439458.1 | unc-80 homolog (C. elegans) |
| RAB5C | ENST00000346213.4 | RAB5C, member RAS oncogene family |
| ABL2 | ENST00000502732.1 | c-abl oncogene 2, non-receptor tyrosine kinase |
| ZNF385A | ENST00000551109.1 | zinc finger protein 385A |
| SYT9 | ENST00000318881.6 | synaptotagmin IX |
| IMPG1 | ENST00000369963.3 | interphotoreceptor matrix proteoglycan 1 |
| TOB2 | ENST00000327492.3 | transducer of ERBB2, 2 |
| SYAP1 | ENST00000380155.3 | synapse associated protein 1 |
| FAM73A | ENST00000370791.3 | family with sequence similarity 73, member A |
| CUL4B | ENST00000371322.5 | cullin 4B |
| ABCC1 | ENST00000399408.2 | ATP-binding cassette, sub-family C (CFTR/MRP), member 1 |
| ANK2 | ENST00000357077.4 | ankyrin 2, neuronal |
| RAB11FIP4 | ENST00000325874.8 | RAB11 family interacting protein 4 (class II) |
| WDTC1 | ENST00000319394.3 | WD and tetratricopeptide repeats 1 |
| FBN2 | ENST00000262464.4 | fibrillin 2 |
| PDE7A | ENST00000401827.3 | phosphodiesterase 7A |
| DCUN1D4 | ENST00000334635.5 | DCN1, defective in cullin neddylation 1, domain containing 4 |
| TMEM33 | ENST00000504986.1 | transmembrane protein 33 |
| KMT2C | ENST00000262189.6 | lysine (K)-specific methyltransferase 2C |
| ZBTB16 | ENST00000335953.4 | zinc finger and BTB domain containing 16 |
| NFIB | ENST00000397575.3 | nuclear factor I/B |
| LIN28B | ENST00000345080.4 | lin-28 homolog B (C. elegans) |
| SPTBN1 | ENST00000356805.4 | spectrin, beta, non-erythrocytic 1 |
| KIAA1549 | ENST00000440172.1 | KIAA1549 |
| TRAF3IP2 | ENST00000368761.5 | TRAF3 interacting protein 2 |
| MPPED1 | ENST00000417669.2 | metallophosphoesterase domain containing 1 |
| VPS4B | ENST00000238497.5 | vacuolar protein sorting 4 homolog B (S. cerevisiae) |
| ENTPD7 | ENST00000370489.4 | ectonucleoside triphosphate diphosphohydrolase 7 |
| VTI1B | ENST00000554659.1 | vesicle transport through interaction with t-SNAREs 1B |
| NMT1 | ENST00000592782.1 | N-myristoyltransferase 1 |
| HMGXB3 | ENST00000503427.1 | HMG box domain containing 3 |
| MED8 | ENST00000372457.4 | mediator complex subunit 8 |
| PARVA | ENST00000334956.8 | parvin, alpha |
| NFIA | ENST00000403491.3 | nuclear factor I/A |
| STARD13 | ENST00000336934.5 | StAR-related lipid transfer (START) domain containing 13 |
| UCK2 | ENST00000372212.4 | uridine-cytidine kinase 2 |
| ARHGAP24 | ENST00000395184.1 | Rho GTPase activating protein 24 |
| FAM102B | ENST00000370035.3 | family with sequence similarity 102, member B |
| CYLD | ENST00000540145.1 | cylindromatosis (turban tumor syndrome) |
| TNRC6B | ENST00000335727.9 | trinucleotide repeat containing 6B |
| SLC30A3 | ENST00000233535.4 | solute carrier family 30 (zinc transporter), member 3 |
| RP1-170O19.20 | ENST00000470747.4 | Uncharacterized protein |
| CCNT1 | ENST00000261900.3 | cyclin T1 |
| ARHGDIA | ENST00000269321.7 | Rho GDP dissociation inhibitor (GDI) alpha |
| LUZP1 | ENST00000418342.1 | leucine zipper protein 1 |
| HOXA9 | ENST00000396345.1 | homeobox A9 |
| BNC2 | ENST00000380672.4 | basonuclin 2 |
| ZNF354B | ENST00000322434.3 | zinc finger protein 354B |
| TNIK | ENST00000436636.2 | TRAF2 and NCK interacting kinase |
| PPP1R9B | ENST00000316878.6 | protein phosphatase 1, regulatory subunit 9B |
| ADCY5 | ENST00000462833.1 | adenylate cyclase 5 |
| PTPRT | ENST00000373187.1 | protein tyrosine phosphatase, receptor type, T |
| SP1 | ENST00000426431.2 | Sp1 transcription factor |
| AAK1 | ENST00000409085.4 | AP2 associated kinase 1 |
| GIGYF1 | ENST00000275732.5 | GRB10 interacting GYF protein 1 |
| BHLHE41 | ENST00000242728.4 | basic helix-loop-helix family, member e41 |
| CAPN5 | ENST00000531028.1 | calpain 5 |
| CHD2 | ENST00000394196.4 | chromodomain helicase DNA binding protein 2 |
| CGGBP1 | ENST00000309534.6 | CGG triplet repeat binding protein 1 |
| SF3B1 | ENST00000335508.6 | splicing factor 3b, subunit 1, 155kDa |
| NFAT5 | ENST00000354436.2 | nuclear factor of activated T-cells 5, tonicity-responsive |
| ID4 | ENST00000378700.3 | inhibitor of DNA binding 4, dominant negative helix-loop-helix protein |
| OTUD4 | ENST00000454497.2 | OTU domain containing 4 |
| TGOLN2 | ENST00000377386.3 | trans-golgi network protein 2 |
| CNNM4 | ENST00000540067.1 | cyclin M4 |
| LEPROTL1 | ENST00000321250.8 | leptin receptor overlapping transcript-like 1 |
| USP32 | ENST00000300896.4 | ubiquitin specific peptidase 32 |
| FAM208A | ENST00000493960.2 | family with sequence similarity 208, member A |
| ARRB1 | ENST00000420843.2 | arrestin, beta 1 |
| FAM46C | ENST00000369448.3 | family with sequence similarity 46, member C |
| ATRX | ENST00000373344.5 | alpha thalassemia/mental retardation syndrome X-linked |
| PROX1 | ENST00000366958.4 | prospero homeobox 1 |
| MKL2 | ENST00000318282.5 | MKL/myocardin-like 2 |
| NIPA1 | ENST00000337435.4 | non imprinted in Prader-Willi/Angelman syndrome 1 |
| SH3PXD2B | ENST00000311601.5 | SH3 and PX domains 2B |
| ADCYAP1 | ENST00000579794.1 | adenylate cyclase activating polypeptide 1 (pituitary) |
| DMXL1 | ENST00000311085.8 | Dmx-like 1 |
| PITPNM3 | ENST00000421306.3 | PITPNM family member 3 |
| SRSF10 | ENST00000343255.5 | serine/arginine-rich splicing factor 10 |
| TRAF3 | ENST00000560371.1 | TNF receptor-associated factor 3 |
| ICK | ENST00000350082.5 | intestinal cell (MAK-like) kinase |
| ARNTL2 | ENST00000546179.1 | aryl hydrocarbon receptor nuclear translocator-like 2 |
| NUP153 | ENST00000262077.2 | nucleoporin 153kDa |
| HECW1 | ENST00000395891.2 | HECT, C2 and WW domain containing E3 ubiquitin protein ligase 1 |
| SFMBT2 | ENST00000361972.4 | Scm-like with four mbt domains 2 |
| MFSD6 | ENST00000392328.1 | major facilitator superfamily domain containing 6 |
| PAWR | ENST00000328827.4 | PRKC, apoptosis, WT1, regulator |
| CAMK1D | ENST00000378847.3 | calcium/calmodulin-dependent protein kinase ID |
| PANK3 | ENST00000239231.6 | pantothenate kinase 3 |
| PCDH1 | ENST00000503492.1 | protocadherin 1 |
| SPTLC2 | ENST00000216484.2 | serine palmitoyltransferase, long chain base subunit 2 |
| FOXK2 | ENST00000335255.5 | forkhead box K2 |
| DDX3X | ENST00000399959.2 | DEAD (Asp-Glu-Ala-Asp) box helicase 3, X-linked |
| RRP15 | ENST00000366932.3 | ribosomal RNA processing 15 homolog (S. cerevisiae) |
| RLN2 | ENST00000308420.3 | relaxin 2 |
| ATP2A2 | ENST00000395494.2 | ATPase, Ca++ transporting, cardiac muscle, slow twitch 2 |
| SRPK1 | ENST00000373825.2 | SRSF protein kinase 1 |
| ISCA2 | ENST00000554924.1 | iron-sulfur cluster assembly 2 |
| RTN4RL1 | ENST00000331238.6 | reticulon 4 receptor-like 1 |
| LPHN1 | ENST00000340736.6 | latrophilin 1 |
| HIC2 | ENST00000407464.2 | hypermethylated in cancer 2 |
| PPM1H | ENST00000228705.6 | protein phosphatase, Mg2+/Mn2+ dependent, 1H |
| MCL1 | ENST00000369026.2 | myeloid cell leukemia sequence 1 (BCL2-related) |
| ZHX3 | ENST00000309060.3 | zinc fingers and homeoboxes 3 |
| ADARB2 | ENST00000381312.1 | adenosine deaminase, RNA-specific, B2 (non-functional) |
| MECP2 | ENST00000303391.6 | methyl CpG binding protein 2 (Rett syndrome) |
| FOXP1 | ENST00000318789.4 | forkhead box P1 |
| FBXL19 | ENST00000338343.4 | F-box and leucine-rich repeat protein 19 |
| CALM1 | ENST00000356978.4 | calmodulin 1 (phosphorylase kinase, delta) |
| DESI1 | ENST00000263256.6 | desumoylating isopeptidase 1 |
| ACAT2 | ENST00000541436.1 | acetyl-CoA acetyltransferase 2 |
| ZBTB7B | ENST00000368426.3 | zinc finger and BTB domain containing 7B |
| FARP1 | ENST00000595437.1 | FERM, RhoGEF (ARHGEF) and pleckstrin domain protein 1 (chondrocyte-derived) |
| ESYT2 | ENST00000251527.5 | extended synaptotagmin-like protein 2 |
| SRGAP3 | ENST00000383836.3 | SLIT-ROBO Rho GTPase activating protein 3 |
| WIPF2 | ENST00000323571.4 | WAS/WASL interacting protein family, member 2 |
| LGALS8 | ENST00000526589.1 | lectin, galactoside-binding, soluble, 8 |
| FST | ENST00000396947.3 | follistatin |
| SLC8A1 | ENST00000406785.2 | solute carrier family 8 (sodium/calcium exchanger), member 1 |
| BNIP3L | ENST00000380629.2 | BCL2/adenovirus E1B 19kDa interacting protein 3-like |
| KCTD16 | ENST00000507359.3 | potassium channel tetramerization domain containing 16 |
| SESN3 | ENST00000536441.1 | sestrin 3 |
| UNKL | ENST00000389221.4 | unkempt homolog (Drosophila)-like |
| FOXN3 | ENST00000345097.4 | forkhead box N3 |
| BAMBI | ENST00000375533.3 | BMP and activin membrane-bound inhibitor |
| PIP4K2B | ENST00000269554.3 | phosphatidylinositol-5-phosphate 4-kinase, type II, beta |
| RPAP2 | ENST00000610020.1 | RNA polymerase II associated protein 2 |
| CHD3 | ENST00000380358.4 | chromodomain helicase DNA binding protein 3 |
| CSTF2 | ENST00000415585.2 | cleavage stimulation factor, 3' pre-RNA, subunit 2, 64kDa |
| SPTY2D1 | ENST00000336349.5 | SPT2, Suppressor of Ty, domain containing 1 (S. cerevisiae) |
| CEP128 | ENST00000281129.3 | centrosomal protein 128kDa |
| PRMT6 | ENST00000370078.1 | protein arginine methyltransferase 6 |
| ZNF322 | ENST00000415922.2 | zinc finger protein 322 |
| ORC4 | ENST00000392857.5 | origin recognition complex, subunit 4 |
| AQP1 | ENST00000509504.1 | Uncharacterized protein |
| ZBTB20 | ENST00000462705.1 | zinc finger and BTB domain containing 20 |
| ENPP6 | ENST00000296741.2 | ectonucleotide pyrophosphatase/phosphodiesterase 6 |
| TG | ENST00000519543.1 | thyroglobulin |
| MAP7 | ENST00000354570.3 | microtubule-associated protein 7 |
