# Supplementary material for: Transforming growth factor (TGF)-β1-induced miR-133a inhibits myofibroblast differentiation and pulmonary fibrosis
Source: Cell Death Dis. 2019 Sep 11;10(9):670. doi: 10.1038/s41419-019-1873-x (PMC6739313; doi:10.1038/s41419-019-1873-x)
Supplement: Supplementary file 6 — Supplementary figure legends. [file 41419_2019_1873_MOESM6_ESM.docx]

**Supplemental Figure legends**

**Supplemental Fig. 1:** **Upregulation of miR-133a is TGF-β1 specific.**

HFL cells were stimulated without or with 1 ng/mL TGF-β1 or 10 ng/mL TNF-α for 48 h, and RNA was harvested and subject to quantitative RT-PCR analysis of miR-133a. Expression levels are presented as the mean ± SEM, n = 3, ****P* < 0.001.

**Supplemental Fig. 2:** **TGF-β1-induced upregulation of miR-133a is independent of PI3K/AKT pathway.**

HFL cells were pretreated with 10 µM of p38MAPK inhibitor (SB203580), PI3K inhibitor (LY294002) or vehicle (DMSO) for 30 min, and then stimulated with 1 ng/mL of TGF-β1 for 48 h. Cells were harvested and subjected to western blot analysis of α-SMA and CTGF protein expression **(a)** or quantitative RT-PCR analysis of miR-133a expression **(b)**. Data are presented as the mean ± SEM, n = 3, **P* < 0.05 and ****P* < 0.001.

**Supplemental Fig. 3: MiR-1 does not alter the TGF-β1 profibrogenic pathway**.

HFL cells were transfected with 30 nM of miR-1 or control mimic (CTL) for 24 h, and then were stimulated without or with 1 ng/mL TGF-β1 for 48 h. TGFBR1 and CTGF protein expression levels were analyzed by western blot **(a)** and GAPDH was used as an internal control. Col1a1 and Col4a1 mRNA expression levels were analyzed by quantitative RT-PCR **(b)**. Experiments were performed three times. Data are mean ± SEM (n=3), ns: not significant.
